# Colorectal Cancer Cell's Weapon: RNF32 Engages SPP1^+^ Macrophages to Foster Liver Metastasis, Targeted by Indole‐3‐Acetic Acid

**DOI:** 10.1002/advs.202519735

**Published:** 2025-12-12

**Authors:** Hongyu Wang, Shipeng Dai, Yuchen Xie, Pengyu Chen, Yue Chai, Chongyu Wang, Xueying Huang, Xiao Dong, Junfeng Shi, Yongxiang Xia, Xiaofeng Qian, Weiwei Tang, Yichan Zhou

**Affiliations:** ^1^ Hepatobiliary Center The First Affiliated Hospital of Nanjing Medical University Key Laboratory of Liver Transplantation Chinese Academy of Medical Sciences, NHC Key laboratory of Hepatobiliary cancers Nanjing 210036 China; ^2^ Department of Geriatric Gastroenterology The First Affiliated Hospital of Nanjing Medical University Nanjing 210036 China; ^3^ Department of Oncology Nanjing First Hospital Nanjing Medical University Nanjing 210006 China

**Keywords:** colorectal cancer liver metastasis, epithelial‐mesenchymal transition, indole‐3‐acetic acid, RNF32, SPP1^+^ macrophage

## Abstract

Colorectal cancer liver metastasis (CRLM) involves complex molecular mechanisms. By integrating The Cancer Genome Atlas (TCGA) data and employing Cox regression, Weighted Gene Co‐expression Network Analysis (WGCNA), and single‐cell RNA sequencing, this study identifies RNF32 as a key gene linking poor prognosis to metastasis. Functional assays demonstrate that RNF32 promotes tumor cell proliferation, invasion, and epithelial–mesenchymal transition (EMT) in vitro, and drives tumor growth and liver metastasis in vivo. Mechanistically, RNF32 catalyzes K48‐linked ubiquitination at the K60 site of GSK3β, stabilizing β‐catenin and activating the Wnt signaling pathway, thereby upregulating CCL2. Mass cytometry and other experiments further reveal that RNF32 recruits SPP1^+^ macrophages via CCL2 to remodel the metastatic niche, a process dependent on the CCR2/FABP1/PPARG axis. Macrophage depletion abrogates metastasis, while the FABP1 inhibitor orlistat reverses SPP1 upregulation in macrophages. Moreover, SPP1^+^ macrophages interact with tumor cell CD44, synergizing with RNF32 to enhance cancer stemness via Wnt signaling. Importantly, virtual screening identifies indole‐3‐acetic acid (IAA) as an RNF32 inhibitor that suppresses liver metastasis and reverses immunosuppression in vivo. This study establishes RNF32 as a dual‐functional driver of metastasis and proposes IAA as a promising therapeutic agent, offering new hope for targeting both tumor‐intrinsic EMT and the immune microenvironment in CRC liver metastasis.

## Graphical abstract

Cytoplasmic RNF32 fuels CRC liver metastasis by degrading GSK3β, which stabilizes β‐catenin and activates Wnt/EMT. Moreover, RNF32 rewires the metastatic niche: it depletes CD8+/CD4+ T and NK cells while recruiting SPP1+ macrophages (which boost tumor stemness via CD44), fibroblasts, and immunosuppressive monocytes to aid colonization. Importantly, indole‐3‐acetic acid (IAA) counteracts RNF32, reversing tumor progression and immunosuppression.

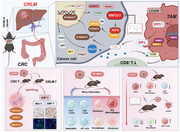

## 1 Introduction

Colorectal cancer (CRC) represents a significant global health challenge, ranking as the third most commonly diagnosed cancer and the second leading cause of cancer‐related deaths worldwide. Recent epidemiological data from 2022 indicate a substantial disease burden, with over 1.9 million new cases and ≈904 000 fatalities.^[^ ^1^ ^]^ Notably, emerging trends highlight a concerning rise in CRC incidence among younger populations, particularly those under 50 years of age. Projections estimate dramatic increases of 90.0% for colon cancer and 124.2% for rectal cancer among individuals aged 20–34 years.^[^ ^2^, ^3^ ^]^ The pathogenesis of CRC involves several well‐established risk factors, including inflammatory bowel disease, tobacco use, dietary habits (especially high intake of red and processed meats), excessive alcohol consumption, diabetes, physical inactivity, metabolic syndrome, and obesity.^[^ ^1^, ^4^ ^]^ Current CRC treatment strategies are tailored according to disease stage and characteristics. For localized disease—encompassing malignant colorectal polyps and resectable non‐metastatic tumors—primary interventions include endoscopic polypectomy and surgical resection. In cases with advanced disease features or a high risk of recurrence, adjuvant or neoadjuvant chemotherapy is commonly integrated into the treatment regimen.^[^ ^5^ ^]^ Despite progress in early detection and therapy, metastatic progression remains a major clinical obstacle. Epidemiological studies report that 50–60% of CRC patients develop metastatic disease, with nearly 80% of these cases involving unresectable liver metastases.^[^ ^6^, ^7^ ^]^ The liver is the most frequent site of metastasis, often developing metachronously after primary tumor treatment.^[^ ^8^ ^]^ Postmortem analyses indicate that more than half of CRC‐related deaths are associated with hepatic metastases. The prognosis for metastatic CRC remains poor, particularly among patients ineligible for surgical intervention, who exhibit significantly lower 5‐year survival rates.^[^ ^6^, ^9^ ^]^ These realities emphasize the urgent need for novel therapeutic approaches, especially for metastatic CRC, which continues to be a critical focus in oncology research.

Gene therapy holds promise for addressing the underlying mechanisms of CRLM; however, its clinical translation remains constrained by challenges in systematic target screening, drug delivery, tumor heterogeneity, and safety. In this study, we integrated TCGA‐based bioinformatics analysis with single‐cell RNA sequencing data to identify RNF32 as a key gene associated with CRC liver metastasis, specifically enriched in tumor cells. As a member of the RING finger (RNF) protein family characterized by a zinc‐coordinating RING domain, RNF proteins play crucial roles in ubiquitination‐dependent processes such as protein degradation, DNA repair, and signal transduction.^[^ ^10^ ^]^ Growing evidence implicates dysregulated RNF family members in tumorigenesis and metastasis across various cancers.^[^ ^11^ ^]^ RNF32, the most recently identified subfamily member, is a unique E3 ubiquitin ligase containing a double RING‐H2 domain and was initially linked to spermatogenesis.^[^ ^12^ ^]^ It has been reported that RNF32 expression is significantly elevated in Barrett's esophagus compared to normal esophageal tissue, and further increased in esophageal adenocarcinoma, suggesting its role in malignant progression.^[^ ^13^ ^]^ A recent bioinformatics and in vitro study indicated that RNF32 may promote macrophage M2 polarization and enhance colon cancer proliferation, migration, and invasion.^[^ ^14^ ^]^ Nevertheless, the functional role and molecular mechanisms of RNF32 in CRC progression and liver metastasis remain poorly understood.

Here, we employed an integrated multi‐omics and experimental approach to establish RNF32 as a central regulator of CRC liver metastasis. We demonstrated that the △2 domain (208–293 aa) of RNF32 directly binds to the D3 domain (281–420 aa) of GSK3β and mediates K48‐linked ubiquitination at the K60 site of GSK3β, leading to activation of the Wnt/β‐catenin pathway. This activation drives epithelial–mesenchymal transition (EMT) and upregulates CCL2 expression, which in turn activates the CCR2/FABP1/PPARG axis in macrophages and induces SPP1 transcription. As a result, SPP1^+^ macrophages are recruited into the tumor microenvironment, fostering a pro‐metastatic niche. Reciprocally, these macrophages feedback to tumor cells via the SPP1/CD44 axis to enhance cancer stemness. This collaborative interaction between tumor cells and SPP1^+^ macrophages accelerates CRC liver metastasis. Targeting this axis, we identified indole‐3‐acetic acid (IAA) as an RNF32 inhibitor that effectively suppresses CRC growth and liver metastasis in vivo and in vitro, demonstrating favorable biosafety profiles.

## 2 Results

### 2.1 Unraveling the Threads: RNF32 Emerges as the Mastermind of Colorectal Cancer Liver Metastasis

**Figure 1:**
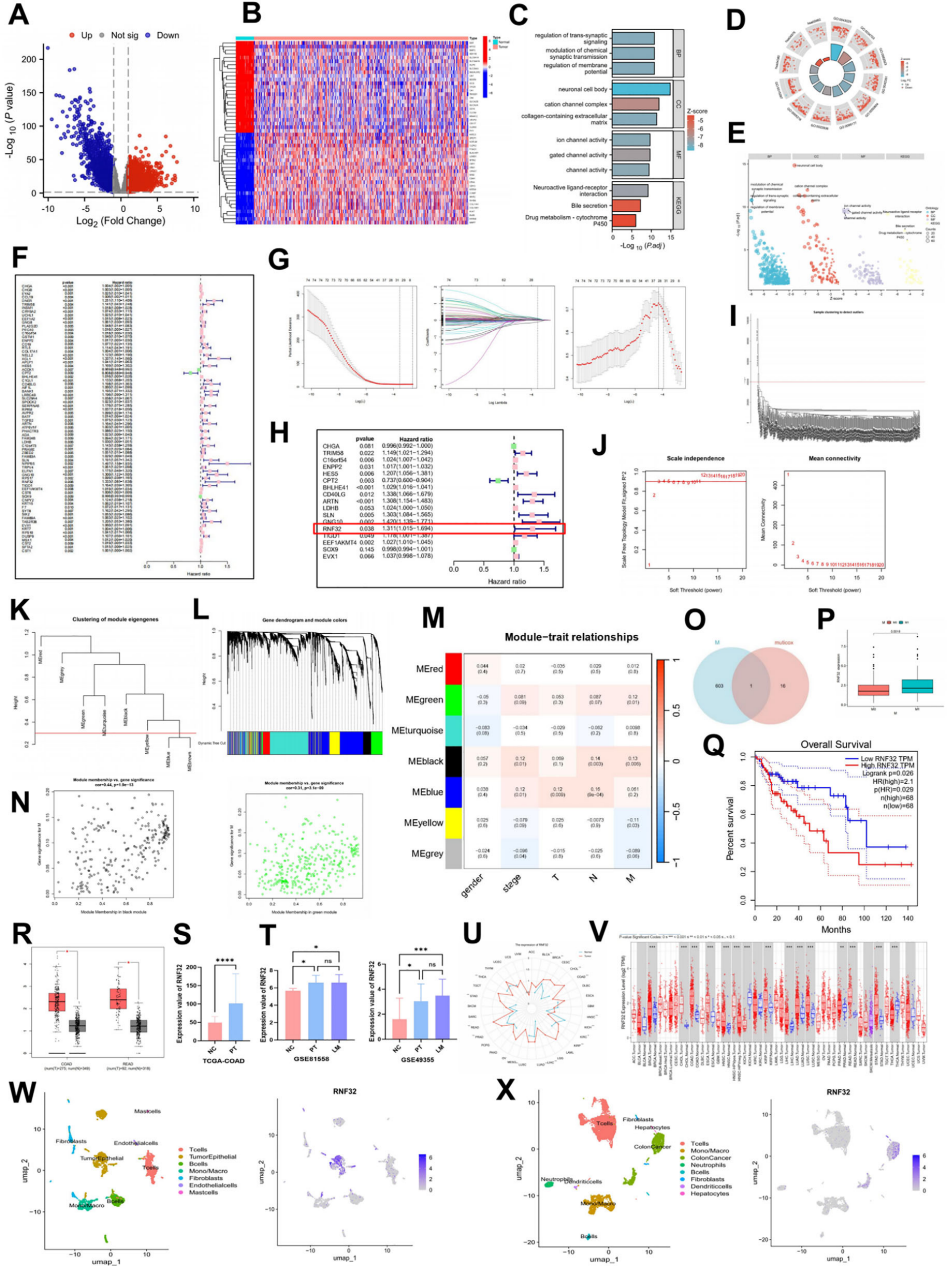
Key genes and pathways associated with liver metastasis in CRC are aggregated based on TCGA data. A,B) The heatmap and volcano map showed differentially expressed genes from the TCGA COAD cohort. C–E) GO analysis revealed that DEGs were significantly enriched in biological processes (BP) such as regulation of trans‐synaptic signaling, cellular components (CC) like neuronal cell body, and molecular functions (MF) including ion channel activity. KEGG pathway enrichment analysis indicated significant associations for the DEGs with Neuroactive ligand‐receptor interaction, Bile secretion, and Drug metabolism‐cytochrome P450, among others. F) The univariate Cox regression analysis of the DEGs in the TCGA COAD cohort. G) The results of the univariate Cox regression were analyzed using LASSO regression. H) Further multivariate Cox regression analysis was conducted on the results of the LASSO regression. I–N) WGCNA was used to analyze DEGs in the TCGA COAD cohort to identify genes associated with CRLM. O) The Venn diagrams of Multifactor Cox regression results and modules related to M stage in WGCNA results. The mRNA expression levels of RNF32 in stage M0 and stage M1 in the TCGA COAD cohort. The prognostic value (*p* < 0.05) of RNF32 in CRC patients in the OS curve (GEPIA). The mRNA expression levels of RNF32 in COAD and READ in the GEPIA database. The mRNA expression level of RNF32 in COAD from the TCGA database. The mRNA expression levels of RNF32 in GSE81558 and GSE49355. V) Results of pan‐cancer analysis of RNF32 in different cancer types with their paracancerous tissues by TIMER database. W,X) UMAP visualizes the cell types of the primary lesion of colorectal cancer and liver metastases, as well as the heterogeneity of RNF32 in different subgroups. In all statistical plots, data are expressed as the mean ± SD, Student's *t*‐test (P,S) One‐way ANOVA (T) were used to determine statistical significance. (ns = not significant, *p < 0.05, ***p < 0.001, ****p < 0.0001).

Differential gene expression analysis comparing 483 colon tumor samples and 41 normal tissues from the TCGA dataset, using thresholds of adjusted p‐value < 0.05 and |log_2_FC| > 1, identified 5093 differentially expressed genes (DEGs), including 2509 upregulated and 2584 downregulated genes in tumors (**Figure** **1**A, Table S1, Supporting Information). A heatmap of the top 50 DEGs is shown in Figure 1B. Functional annotation revealed that these DEGs were significantly enriched in multiple biological domains: Gene Ontology (GO) analysis highlighted their involvement in trans‐synaptic signaling regulation (biological process), localization to neuronal cell bodies (cellular component), and ion channel activity (molecular function). Kyoto Encyclopedia of Genes and Genomes (KEGG) pathway analysis further indicated significant enrichment in Neuroactive ligand–receptor interactions, Bile secretion, and Drug metabolism–cytochrome P450 pathways (Figure 1C–E). Univariate Cox regression (*p* < 0.01) of the 5093 DEGs for overall survival (OS), based on clinical data from 309 colon cancer patients (excluding those with missing survival data and OS ≤ 30 days, Table S2, Supporting Information), identified 74 OS‐related DEGs (Figure 1F). Subsequent LASSO regression (Figure 1G) and multivariate Cox analysis narrowed these down to 17 genes independently associated with prognosis (Figure 1H). In parallel, Weighted Gene Co‐expression Network Analysis (WGCNA) was applied to the 5093 DEGs to identify metastasis‐related genes. After outlier removal (cutHeight = 40 000; Figure 1I) and soft‐thresholding power selection (β = 3; Figure 1J), a hierarchical clustering tree was constructed (Figure 1K), and module merging (cutHeight = 0.3) yielded 7 distinct modules (Figure 1L). Correlation analysis between modules and clinical traits (gender, stage, TNM stage) is visualized in a heatmap (Figure 1M). Only the black and green modules (604 genes total) showed significant correlation with M stage (*p* < 0.05) and were selected; Figure 1N illustrates the correlation between the genes in these two modules and the M stage. Intersecting the 17 prognosis‐related genes with the 604 M‐stage‐related genes identified RNF32 as the overlapping gene (Figure 1O). Validation using TCGA and GEPIA2 confirmed significantly higher RNF32 expression in metastatic (M1 stage) colon cancer (Figure 1P) and poorer OS in patients with high RNF32 expression (p = 0.026; Figure 1Q), supporting its association with poor prognosis and metastasis. RNF32 was also elevated in primary colorectal tumors (COAD, READ) compared to normal tissues in GEPIA (Figure 1R) and TCGA (COAD)(Figure 1S). Analysis of GEO datasets (GSE81558, GSE49355) showed higher RNF32 expression in primary tumors and liver metastases than in normal tissue (Figure 1T, Table S3, Supporting Information). Pan‐cancer analysis via TIMER revealed significant RNF32 upregulation in multiple cancers, especially COAD (Figure 1U, V). Single‐cell sequencing of CRC samples classified primary lesions into 7 cell clusters and CRLM into 8 clusters (UMAP and dot plots in Figure 1W–X and Figure S1A,B, Supporting Information). RNF32 was predominantly enriched in tumor cells from both primary and metastatic sites (Figure 1W–X, Figure S1E,F, Supporting Information). Immunofluorescence confirmed high RNF32 expression in tumor tissues with co‐staining for CK8 (Figure S1C, Supporting Information), and high RNF32 levels correlated with poor prognosis (Figure S1D, Supporting Information). qRT‐PCR and Western blot analyses showed elevated RNF32 expression in moderately (SW480, LoVo) and highly (SW620) metastatic CRC cell lines compared to non‐metastatic HCT116 cells (Figure S2A,B, Supporting Information), indicating upregulation in cells with high metastatic potential. Together, these findings suggest that RNF32 plays a role in CRC progression and liver metastasis, highlighting its potential as a therapeutic target.

### 2.2 RNF32 Steals the Spotlight: Promoting In Vitro Proliferation, Invasion, Migration, and EMT in Colorectal Cancer

**Figure 2:**
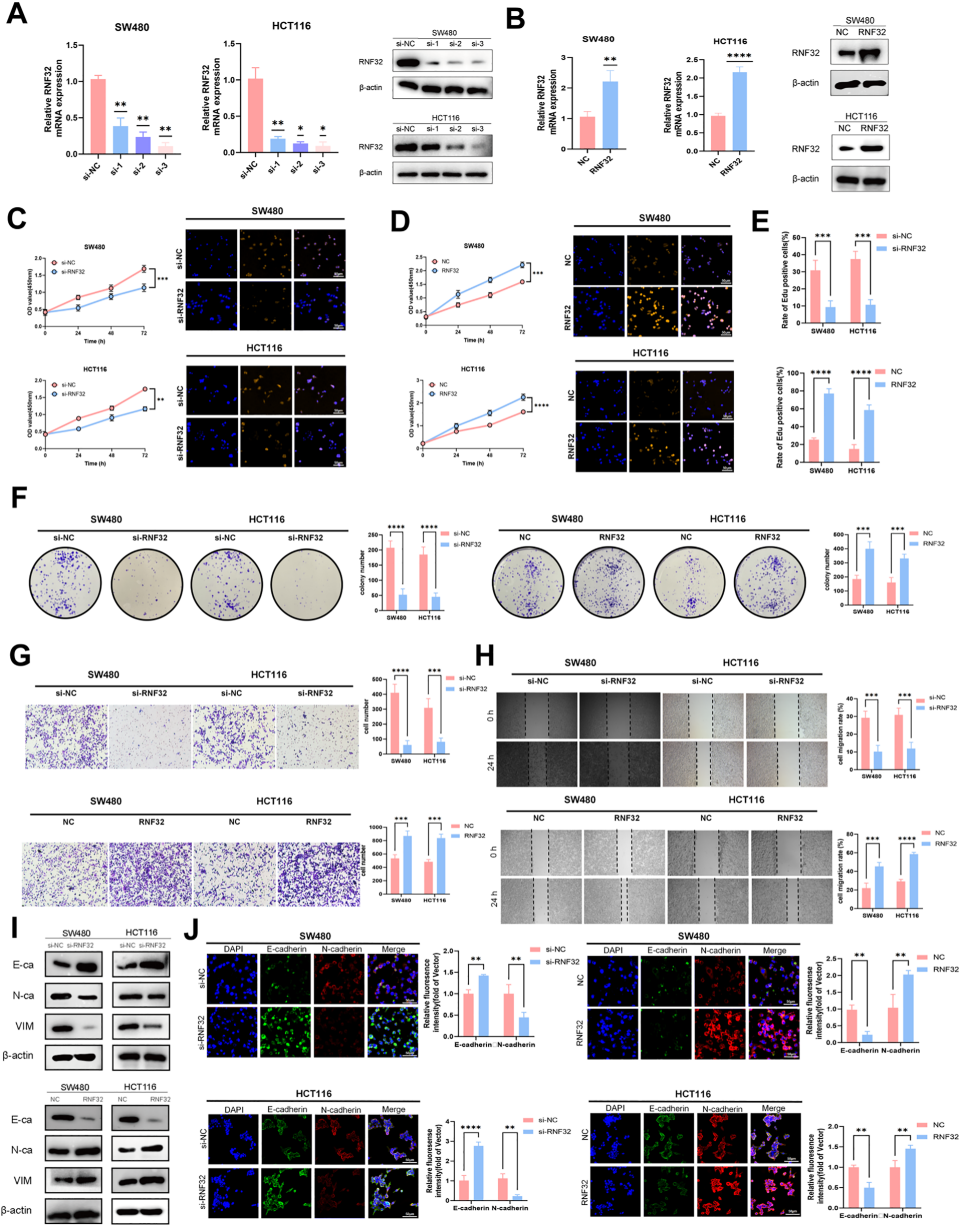
RNF32 promotes proliferation, invasion, migration and EMT of CRC in vitro. A) Relative mRNA and protein expression levels of RNF32 in CRC cell lines after downregulation. n = 3 independent biological replicates. B) Relative mRNA and protein expression levels of RNF32 in CRC cell lines after upregulation. n = 3 independent biological replicates. C–E) EdU assays and CCK‐8 assays were performed to assess the effect of RNF32 on CRC cell proliferation (scale bars, 50 µm). n = 3 independent biological replicates. F) Colony formation assay were carried out to assess the effect of RNF32 on CRC cell proliferation. n = 3 independent biological replicates. G) Transwell assays were performed to determine the invasion capacities of transfected CRC cell lines. n = 3 independent biological replicates. H) The effects of RNF32 on CRC cell migration were evaluated through wound healing assays. n = 3 independent biological replicates. I) Western blot analysis of EMT markers E‐cadherin, N‐cadherin and vimentin in CRC cell lines transfected with RNF32. β‐actin was used as the loading control. n = 3 independent biological replicates. J) Representative immunofluorescence staining of EMT markers E‐cadherin, N‐cadherin and vimentin in CRC cell lines In all statistical plots, data are expressed as the mean ± SD, one‐way ANOVA (A), Student's *t*‐test (B,C,D) and Two‐way ANOVA (E,G,H,J) were used to determine statistical significance. (*p < 0.05, **p < 0.01, ***p < 0.001, ****p < 0.0001).

To elucidate the functional role of RNF32 in CRC, we first evaluated its expression across multiple CRC cell lines. qRT‐PCR and Western blot analyses identified HCT116 and SW480 as exhibiting the lowest and highest RNF32 levels, respectively (Figure S2A,B, Supporting Information), and these lines were therefore selected for subsequent functional studies. Knockdown of RNF32 was achieved by transfecting SW480 and HCT116 cells with control siRNA or three independent RNF32‐targeting siRNAs (si‐1, si‐2, si‐3). Both mRNA and protein levels of RNF32 were significantly reduced upon siRNA transfection, with RNF32 si‐3 showing superior silencing efficiency at both transcriptional and translational levels compared to si‐1 and ‐2, and it was thus chosen for further experiments (**Figure** **2**A). Conversely, RNF32 was overexpressed by transfecting cells with RNF32‐expressing plasmids, while the NC group received empty vector (Figure 2B). Functional assays revealed that RNF32 knockdown markedly impaired cellular proliferation, as evidenced by CCK‐8, EdU, and colony formation assays (Figure 2C–F), whereas RNF32 overexpression enhanced proliferative capacity. Similarly, Transwell assays showed that RNF32 silencing significantly reduced invasive ability after 24 h, while overexpression promoted invasion (Figure 2G). Wound healing assays further indicated impaired migration in RNF32‐depleted cells, an effect that was reversed in RNF32‐overexpressing groups (Figure 2H). At the molecular level, Western blot analysis of EMT markers demonstrated that RNF32 knockdown downregulated mesenchymal markers (N‐cadherin and Vimentin) and upregulated the epithelial marker E‐cadherin, with opposite trends observed in overexpression models (Figure 2I). Immunofluorescence staining corroborated these findings, showing reduced N‐cadherin and Vimentin alongside increased E‐cadherin expression following RNF32 knockdown, which was again reversed by RNF32 overexpression (Figure 2J). Collectively, these results demonstrate that RNF32 facilitates CRC progression by promoting proliferation, invasion, migration, and EMT.

### 2.3 From Dish to Animal: RNF32 Propels Tumor Growth and Metastatic Vigor In Vivo

**Figure 3:**
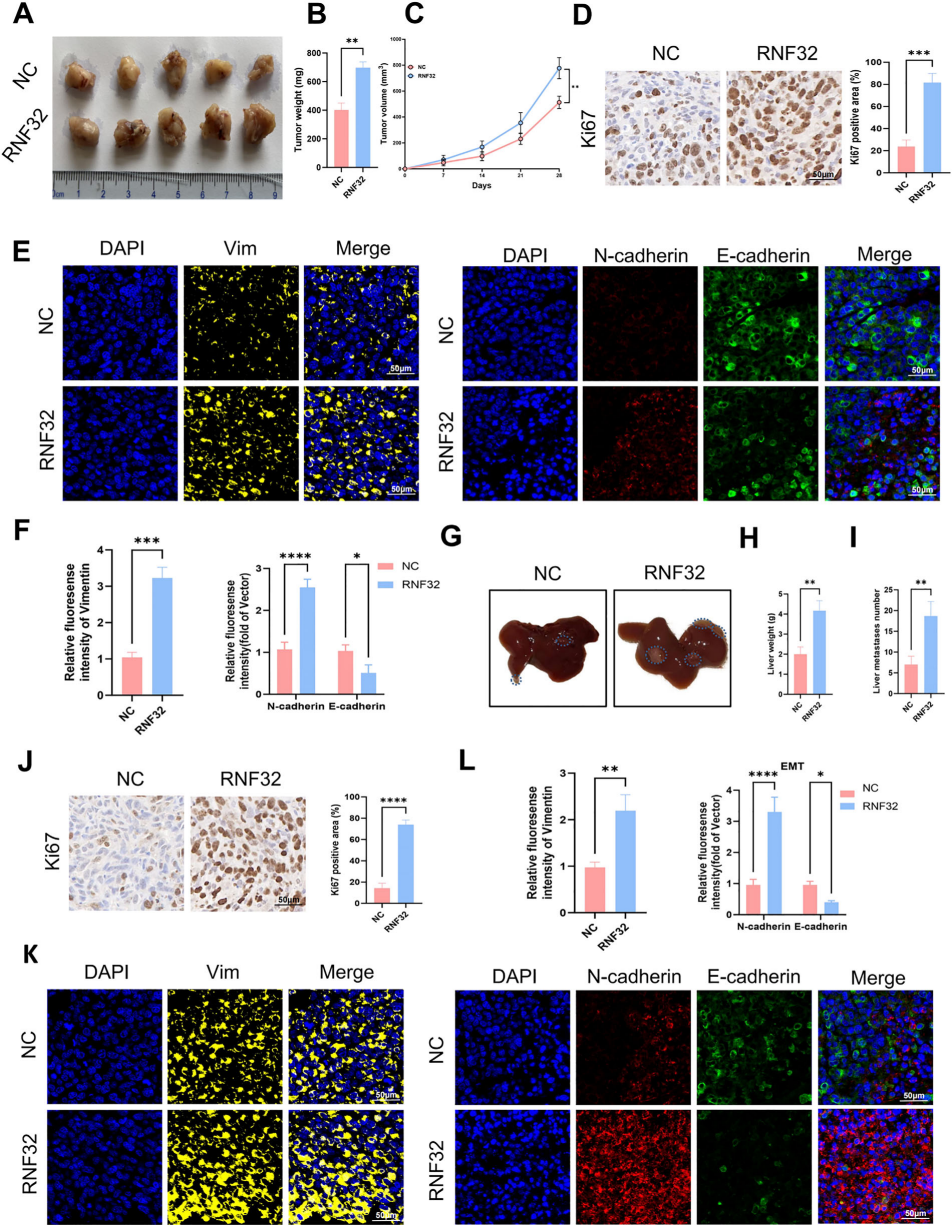
RNF32 promotes CRC growth and liver metastasis in vivo. A) Photographs of xenograft tumors induced by the subcutaneous inoculation of mice (n = 5 mice per group) with transfected MC38 cells. B,C) Statistical plots of tumor weight (B) and tumor volume (C) analysis. The tumor volumes were measured every 7 days, and the tumor weights were analyzed after the mice were sacrificed. D) Representative Ki‐67 immunohistochemical of xenograft tumors (scale bars, 50 µm). n = 3 mice per group. E,F) Representative EMT markers E‐cadherin, N‐cadherin and vimentin Immunofluorescence staining of xenograft tumors (scale bars, 50 µm). n = 3 mice per group. Representative graphs of the livers of mice (n = 5 mice per group) after spleen injection of MC38 cells. The liver weight of the liver metastatic foci of mice (n = 5 mice per group) after spleen injection of MC38 cells. The number of the liver metastatic foci of mice (n = 5 mice per group) after spleen injection of MC38 cells. Representative Ki‐67 immunohistochemical of the liver metastatic foci (scale bars, 50 µm). n = 3 mice per group. K,L) Representative EMT markers E‐cadherin, N‐cadherin and vimentin Immunofluorescence staining of the liver metastatic foci (scale bars, 50 µm). n = 3 mice per group. In all statistical plots, data are expressed as the mean ± SD, Student's *t*‐test (B,C,D,F,H,I,J,L) and One‐way ANOVA (F,L) were used to determine statistical significance. (*p < 0.05, **p < 0.01, ***p < 0.001, ****p < 0.0001).

To further investigate the effects of RNF32 overexpression on tumor growth in vivo, MC38 cells transfected with RNF32‐overexpressing lentivirus or empty vector were subcutaneously injected into the flanks of C57BL/6 mice. The results demonstrated that mice in the RNF32 overexpression group exhibited significantly accelerated subcutaneous tumor growth rates and increased tumor weights compared to the control group (**Figure** **3**A–C). Ki67 staining revealed that tumor cell proliferation was markedly enhanced in the overexpression group (Figure 3D). Immunofluorescence analysis indicated elevated expression of N‐cadherin and Vimentin alongside reduced E‐cadherin levels in RNF32‐overexpressing tumors (Figure 3E,F). Additionally, a spleen‐to‐liver metastasis model was established by intrasplenic injection of modified MC38 cells. 4 weeks post‐injection, livers were harvested for quantification of metastatic foci, followed by immunohistochemical analyses. The RNF32 overexpression group displayed greater numbers of liver metastases with heavier weights relative to controls (Figure 3G–I). Ki67 staining confirmed enhanced proliferative activity in metastatic lesions of the overexpression group (Figure 3J). Consistently, immunofluorescence demonstrated upregulated N‐cadherin and Vimentin expression accompanied by downregulated E‐cadherin in liver metastases from RNF32‐overexpressing groups (Figure 3K,L). Collectively, RNF32 overexpression drives in vivo tumor progression and liver metastasis by enhancing proliferative activity and inducing EMT‐related molecular alterations.

### 2.4 RNF32's Molecular Weapon: Mechanistic Insights into Wnt Pathway Activation

**Figure 4:**
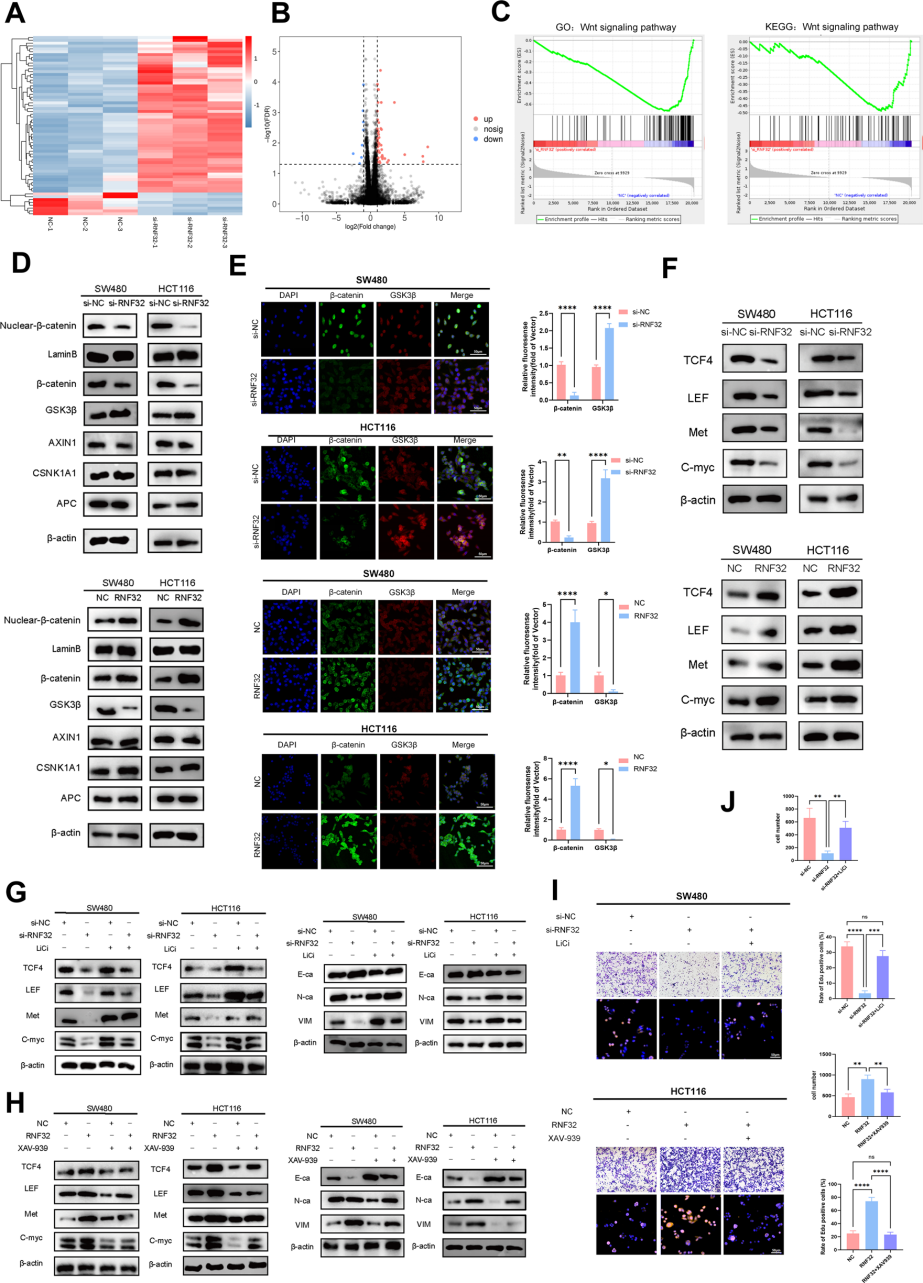
RNF32 drives CRC liver metastasis via GSK3β/Wnt/β‐catenin signaling‐mediated EMT. A,B) The heatmap and volcano map showed differentially expressed genes after RNF32 knockdown in CRC cells. C) GSEA showing that the genes that were differentially expressed after RNF32 knockdown were enriched in the Wnt/β‐catenin signaling gene set. D) Western blot analysis to evaluate proteins related to the Wnt pathway β‐catenin, GSK3β, AXIN1, CSNK1A1 and APC in CRC cell lines transfected with RNF32. β‐actin was used as the loading control of whole‐cell proteins and cytoplasmic proteins. LaminB was used as the loading control of nuclear proteins. n = 3 independent biological replicates. E) Representative β‐catenin and GSK3β Immunofluorescence staining in CRC cell lines transfected with RNF32 (scale bars, 50 µm). n = 3 independent biological replicates. F) Western blot analysis to evaluate Wnt downstream targets TCF4, LEF, Met and c‐Myc in CRC cell lines transfected with RNF32. β‐actin was used as the loading control. n = 3 independent biological replicates. G) Western blot analysis the protein levels of Wnt downstream targets TCF4, LEF, Met and c‐Myc and EMT markers E‐cadherin, N‐cadherin and vimentin in CRC cell lines transfected with or without si‐RNF32, as well as cells treated with or without the Wnt pathway activator LiCl. β‐actin was used as the loading control. n = 3 independent biological replicates. H) Western blot analysis the protein levels of Wnt downstream targets TCF4, LEF, Met and c‐Myc and EMT markers E‐cadherin, N‐cadherin and vimentin in CRC cell lines transfected with or without RNF32, as well as cells treated with or without the Wnt pathway inhibitor XAV939. β‐actin was used as the loading control. n = 3 independent biological replicates. I,J) Transwell and Edu assays were performed to determine the invasion and proliferation capacities of transfected CRC cell lines with or without si‐RNF32 or RNF32, as well as cells treated with or without the Wnt pathway activator LiCl or inhibitor XAV939. In all statistical plots, data are expressed as the mean ± SD, Two‐way ANOVA (E), and One‐way ANOVA (J) were used to determine statistical significance. (ns = not significant, *p < 0.05, **p < 0.01, ***p < 0.001, ****p < 0.0001).

Our above in vitro and vivo experiments preliminarily demonstrated that high RNF32 expression mediates CRC liver metastasis by enhancing invasion and colonization, though its molecular mechanism remained unclear. To investigate how RNF32 regulates EMT and invasion, we performed RNA sequencing in RNF32‐knockdown CRC cells. The results revealed a significant correlation between differential RNF32 expression levels and alterations in Wnt pathway activity (**Figure** **4**A–C; Figure S3A,B, Supporting Information). GSEA of TCGA datasets demonstrated significant upregulation of the Wnt signaling pathway in CRLM compared to primary tumors (Figure S3C, Supporting Information). Spearman's rank analysis of immunofluorescence data from clinical CRC specimens revealed a significant positive correlation between RNF32 and active β‐catenin protein expression (Figure S3D, Supporting Information). Consistently, analysis of TCGA data demonstrated strong positive correlations between RNF32 expression and β‐catenin levels, as well as its downstream targets TCF4, LEF1, and c‐Myc in CRC tissues (Figure S3E,F, Supporting Information). Given that activated β‐catenin drives EMT by regulating ZEB1/ZEB2, and prior reports implicating RNF family members (e.g., RNF4, RNF6, RNF14) in Wnt‐mediated CRC progression,^[^ ^15^, ^16^, ^17^, ^18^, ^19^ ^]^ we hypothesized that RNF32 promotes EMT via the GSK3β/Wnt/β‐catenin axis. Western blotting revealed decreased total β‐catenin levels, reduced nuclear β‐catenin translocation, and elevated total GSK3β expression in RNF32‐knockdown cells, with opposite trends in overexpressing cells (Figure 4D). Immunofluorescence confirmed elevated GSK3β expression and reduced β‐catenin in knockdown groups, with inverse patterns in overexpression groups (Figure 4E). Consistently, protein levels of Wnt downstream targets (TCF4, LEF, Met, c‐Myc) decreased in knockdown cells and increased in overexpressing cells (Figure 4F). To determine whether Wnt activation rescues RNF32 deficiency effects, we treated RNF32‐deficient cells with LiCl (a β‐catenin activator). LiCl restored Wnt/β‐catenin signaling activity, upregulated target genes, suppressed E‐cadherin, and enhanced N‐cadherin/Vimentin expression (Figure 4G). Conversely, the Wnt inhibitor XAV‐939 reversed RNF32 overexpression‐induced upregulation of Wnt targets and EMT markers (Figure 4H). Functional assays demonstrated that LiCl partially rescued the impaired proliferation and invasion of RNF32‐deficient cells, whereas XAV‐939 counteracted RNF32‐driven oncogenic phenotypes (Figure 4I,J). These findings collectively establish RNF32 as a critical regulator of Wnt/β‐catenin signaling in CRC liver metastasis.

### 2.5 Precision Strike: RNF32 Mediates K48‐Linked Ubiquitination at K60 to Direct the Degradation of GSK3β

**Figure 5:**
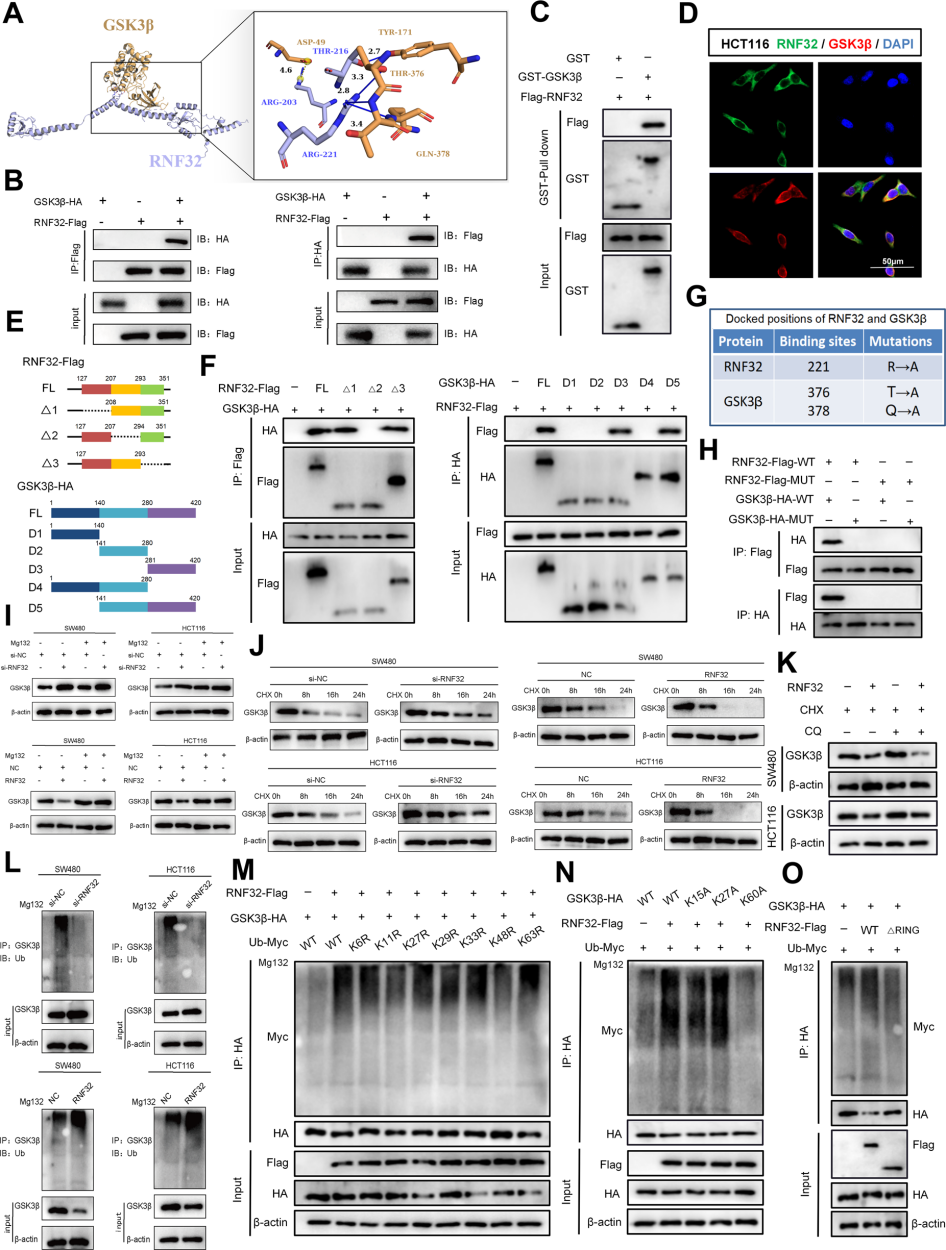
RNF32 Mediates K48‐Linked Ubiquitination at K60 to Direct the Degradation of GSK3β. A) Artificial intelligence (AI) technology was used to docking and found that RNF32 and GSK3β proteins may also have action sites. B) Immunoprecipitation and western blot analysis showing the interaction of RNF32 and GSK3β in CRC cells. C) Analysis of RNF32 and GSK3β interaction in vitro via GST pull‐down assays. Fusion protein beads were employed for pull‐down, and the corresponding proteins labeled with GST/FLAG were detected through WB analysis D) Co‐localization of RNF32 (green) and GSK3β (red) was indicated by immunofluorescence confocal microscopy. DAPI was used for nuclear staining (blue). E) Schematic of RNF32 and GSK3β in full length and truncations. F) Immunoprecipitation and western blot analyses illustrating interactions between FLAG‐tagged truncated RNF32 and HA‐tagged truncated GSK3β proteins in 293T cells. G) Docked positions of RNF32 and GSK3β and mutations of RNF32 and GSK3β interaction sites. H) Immunoprecipitation and western blot analyses showing interactions between FLAG‐tagged mutated RNF32 and HA‐tagged mutated GSK3β in 293T cells I) MG132 (10 µM) contact si‐RNF32 or RNF32 transfected CRC cell lines with 8h. GSK3β protein expression was examined by Western Blotting. n = 3 independent biological replicates. J) Western blot analysis of GSK3β protein in si‐RNF32 or RNF32 transfected CRC cell lines treated with cycloheximide (CHX; 25 µg mL^−1^) for 0, 8, 16, and 24 h. n = 3 independent biological replicates. K) Chloroquine contact RNF32 transfected CRC cell lines. GSK3β protein expression was examined by Western Blotting. n = 3 independent biological replicates. L) At 8 h post‐treatment with MG132 (10 µM), cell lysates were obtained and immunoprecipitated with anti‐GSK3β antibody. Western blot analysis of IPs performed with antibody to GSK3β to detect endogenous GSK3β ubiquitination from si‐RNF32 or RNF32 transfected CRC cell lines. M) Co‐IP assays were applied to assess the ubiquitination level of GSK3β affected by the plasmids containing seven specific ubiquitin mutants. N) The ubiquitination level of GSK3β influenced by the plasmids containing three GSK3β mutants was measured by Co‐IP assays. O) Ubiquitination level of HA‐GSK3β in 293T cells transfected with HA‐GSK3β, Myc‐Ub, and Flag‐RNF32 (WT) or Flag‐RNF32‐ΔRing plasmids.

Our study further explored how RNF32 mechanistically regulates the GSK3β/Wnt/β‐catenin axis. In the absence of Wnt ligands, cytoplasmic β‐catenin is phosphorylated by a destruction complex comprising GSK3β, casein kinase I (CK1), Axin, and adenomatous polyposis coli (APC). GSK3β‐mediated phosphorylation promotes APC‐dependent recruitment of β‐catenin to ubiquitin‐proteasomal degradation.^[^ ^15^ ^]^ As RNF32 is an E3 ubiquitin ligase predominantly localized in the cytoplasm,^[^ ^12^ ^]^ molecular docking predicted a potential interaction between RNF32 and GSK3β (**Figure** **5**A). To investigate the protein–protein interaction between RNF32 and GSK3β, FLAG‐tagged RNF32 and HA‐tagged GSK3β were overexpressed in 293T cells. Co‐immunoprecipitation (Co‐IP) revealed reciprocal binding, with each protein pulling down the other (Figure 5B). A GST pull‐down assay further confirmed their direct interaction (Figure 5C). Endogenous co‐localization of RNF32 and GSK3β was validated by immunofluorescence (IF) in HCT116 cells (Figure 5D). To identify the interacting regions, a series of truncation mutants were designed based on protein domain structures (Figure 5E). Co‐IP assays in 293T cells demonstrated that the △2 domain (208–293 aa) of RNF32 and the D3 domain (281–420 aa) of GSK3β are essential for their interaction (Figure 5F). Based on HADDOCK‐predicted binding residues, we generated point mutations at RNF32 residue 221 and GSK3β residues 376 and 378 (Figure 5G). Co‐IP confirmed that mutating either or both binding sites markedly disrupted the RNF32–GSK3β interaction (Figure 5H). Given that RNF32 is a cytoplasmic E3 ubiquitin ligase, we hypothesized that it targets GSK3β for ubiquitin‐mediated degradation to stabilize β‐catenin. Cycloheximide (CHX) chase assays in RNF32‐knockdown CRC cells showed prolonged GSK3β half‐life, whereas RNF32 overexpression accelerated its decay (Figure 5J). MG132 treatment reversed GSK3β accumulation induced by RNF32 knockdown, but chloroquine did not (Figure 5I,K), supporting proteasomal degradation. Moreover, RNF32 knockdown reduced GSK3β ubiquitination and increased its protein level, while its overexpression had the opposite effect (Figure 5L). In contrast, the mutant form of RNF32 was unable to influence GSK3β ubiquitination (Figure S3G, Supporting Information), demonstrating that a functional RNF32 is required for this regulation. Among seven lysine‐linked ubiquitin chains, Myc‐Ub‐K48R markedly impaired GSK3β ubiquitination, indicating K48 linkage predominance (Figure 5M). Site‐directed mutagenesis of three predicted lysine ubiquitination sites in GSK3β identified K60 as critical for RNF32‐mediated K48‐linked ubiquitination (Figure 5N). Finally, ubiquitination assays showed that wild‐type RNF32, but not a RING‐deleted mutant (ΔRing), promoted GSK3β ubiquitination (Figure 5O). In summary, RNF32 directly binds GSK3β and acts as an E3 ligase that targets K60 for K48‐linked polyubiquitination and proteasomal degradation, thereby exerting essential control over GSK3β protein stability.

### 2.6 A Pact with the Devil: RNF32 Orchestrates a Pro‐Metastatic Niche to Drive Colorectal Cancer Liver Metastasis

**Figure 6:**
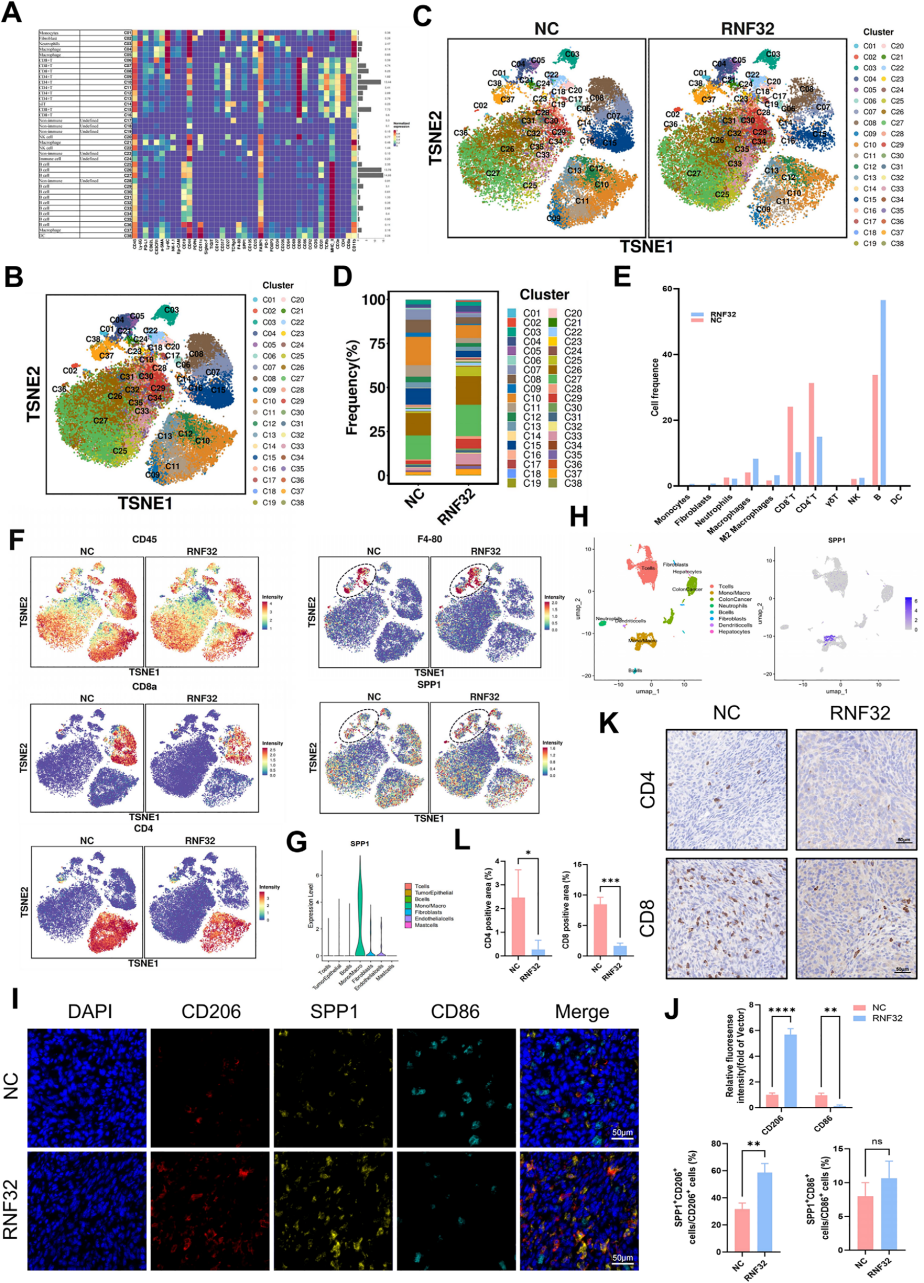
RNF32 overexpression promotes the colonization of CRC cells in the liver by inducing alterations in the tumor microenvironment. A) Mass cytometry analysis of liver metastases post NC/RNF32 injection revealed 38 distinct cell clusters. Single‐cell suspensions were isolated from viable, intact cells in target tissues, enabling comprehensive cell aggregation and subpopulation characterization across all samples. B–E) The TSNE scatter plot showed the distribution of each cell cluster, and the proportion of each cell cluster was statistically analyzed. F) In RNF32‐overexpressing CRC liver metastases, key immune markers such as CD45, CD4, and CD8a were significantly downregulated, while SPP1 expression was elevated in macrophages. G,H) UMAP and Violin plot visualize the cell types of liver metastases, as well as the heterogeneity of SPP1 in different subgroups. I,J) Representative CD206, SPP1 and CD86 immunohistochemical of the liver metastases (scale bars, 50 µm). n = 3 mice per group. K,L) Representative CD4 and CD8 immunofluorescence staining of the liver metastases (scale bars, 50 µm). n = 3 mice per group. In all statistical plots, data are expressed as the mean ± SD, Two‐way ANOVA (J) and Student's *t*‐test (J,L) were used to determine statistical significance. (ns = not significant, *p < 0.05, **p < 0.01, ***p < 0.001, ****p < 0.0001).

While the aforementioned study established that RNF32 promotes CRC liver metastasis through EMT, its specific roles in hepatic colonization and remodeling of the metastatic tumor microenvironment (TME) remain unclear. Immune infiltration analysis of the GEO dataset (GSE81558) demonstrated a marked increase in M2 macrophages in CRC liver metastases compared to primary tumors (Figure S4A, Supporting Information), reinforcing the pivotal involvement of tumor‐associated macrophages (TAMs) in driving hepatic metastasis—a observation consistent with our previous studies on the CRC TME.^[^ ^20^, ^21^ ^]^ To assess the influence of RNF32 expression on the tumor immune landscape, we conducted mass cytometry (CyTOF) on liver metastases from control and RNF32‐overexpressing mouse models. This high‐dimensional analysis identified 38 distinct immune cell clusters, each precisely annotated according to specific marker expression patterns (**Figure** **6**A,B, Figure S4B, Supporting Information). Comparative evaluation revealed substantial shifts in immune cell composition upon RNF32 overexpression (Figure 6C,D). Notably, we detected a significant elevation in the relative proportions of M2 macrophages, fibroblasts, monocytes, and γδ T cells (Figure 6E). In contrast, the abundances of immunostimulatory populations—including CD8⁺ T cells, CD4⁺ T cells, dendritic cells (DCs), and natural killer (NK) cells—were reduced (Figure 6E). Furthermore, in RNF32‐overexpressing CRC liver metastases, key immune markers such as CD45, CD4, and CD8a were significantly downregulated, while SPP1 expression was elevated in macrophages (Figure 6F). Single‐cell data confirmed pronounced enrichment of SPP1 within myeloid cells in metastatic lesions (Figure 6G,H). Immunofluorescence analysis revealed strong co‐localization of SPP1 and CD206 in CRC liver metastases, with RNF32 overexpression significantly increasing the proportion of SPP1⁺ M2 macrophages, but not affecting M1 macrophages (Figure 6I,J). Additionally, immunohistochemistry validated that RNF32 overexpression suppressed CD4 and CD8 expression in metastatic liver tissues(Figure 6K,L). Collectively, these findings suggest that RNF32 facilitates hepatic colonization of CRC cells by orchestrating immunosuppressive alterations in the tumor microenvironment.

### 2.7 A Soul Sold: SPP1+ Macrophages Pledge Allegiance to RNF32 in Colorectal Cancer

**Figure 7:**
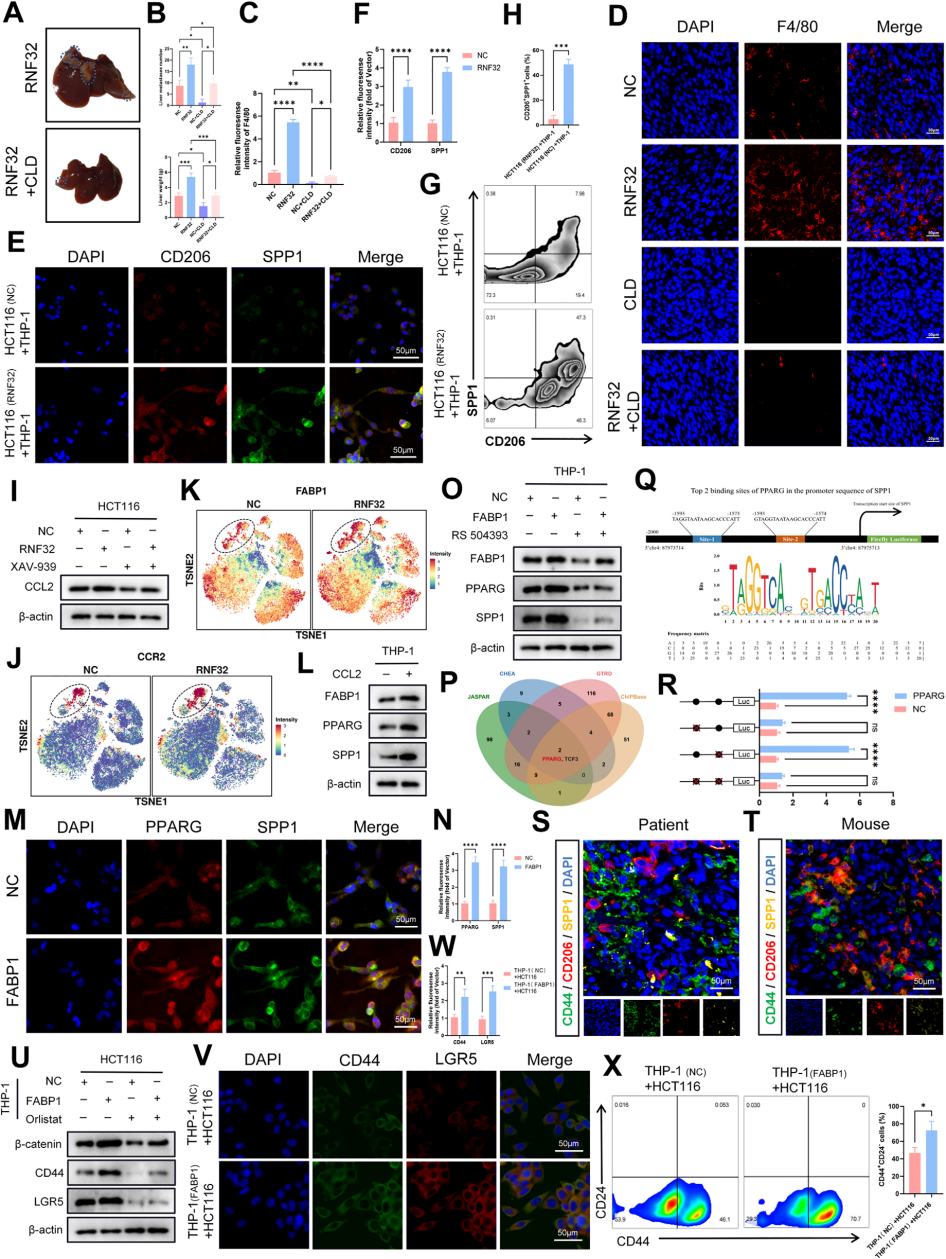
RNF32 drives CRC liver metastasis by promoting SPP1^+^ Macrophage and an immunosuppressive microenvironment. A) Representative graphs of the livers of mice (n = 5 mice per group) after spleen injection of MC38 cells transfected with RNF32, as well as treated with or without CLD. B) The liver weight of the liver metastases of mice (n = 5 mice per group). C) The number of the liver metastases of mice (n = 5 mice per group). D) Representative F4/80 immunofluorescence staining of the liver metastases of mice (n = 5 mice per group) after spleen injection of transfected MC38 cell lines with or without RNF32, as well as cells treated with or without the CLD. E,F) Representative CD206 and SPP1 immunofluorescence staining in THP‐1 cells cultured with conditioned medium from RNF32‐overexpressing CRC cells (scale bars, 50 µm). n = 3 independent biological replicates. G,H) The proportions of CD206^+^ SPP1^+^ cells were analyzed separately by flow cytometry. I) Western blot analysis the protein levels of CCL2 in CRC cell lines transfected with or without RNF32, as well as cells treated with or without the Wnt pathway inhibitor XAV939. β‐actin was used as the loading control. n = 3 independent biological replicates. J,K) The expression of FABP1 and CCR2 was increased in the RNF32 group relative to the NC group. L) Western blot analysis the protein levels of FABP1, PPARG and SPP1 in THP‐1 macrophages added with or without CCL2 (20 mM). β‐actin was used as the loading control. n = 3 independent biological replicates. M,N) Representative CD206 and SPP1 immunofluorescence staining in THP‐1 macrophages transfected with or without FABP1 (scale bars, 50 µm). n = 3 independent biological replicates. O) Western blot analysis the protein levels of FABP1, PPARG and SPP1 in THP‐1 macrophages transfected with or without FABP1, as well as cells treated with or without RS 504393. β‐actin was used as the loading control. n = 3 independent biological replicates. P) Analysis OF Transcription factor databases (JASPAR, CHEA, GTRD, ChIPBase) identified PPARG as one of potential transcription factor driving SPP1 expression. Q) Putative PPARG‐binding sites identified in the promoter region of SPP1 gene. R) Dual‐luciferase reporter assays showing that the site 1 mutation abrogated capacity of PPARG to act on the SPP1 promoter region. The red “X” within the binding regions indicates altered PPARG binding sequences. S,T) Immunofluorescence staining revealed close proximity between SPP1⁺ macrophages and CD44⁺ CSCs within tumors. U) Western blot analysis the protein levels of, β‐catenin, CD44 and LGR5 in HCT116 cells co‐cultured with or without FABP1‐overexpressing THP‐1 macrophages, as well as cells treated with or without Orlistat. β‐actin was used as the loading control. n = 3 independent biological replicates. V,W) Representative CD44 and LGR5 immunofluorescence staining in HCT116 cells co‐cultured with or without FABP1‐overexpressing THP‐1 macrophages (scale bars, 50 µm). n = 3 independent biological replicates. X) The proportions of CD44^+^ CD24^−^ cells were analyzed separately by flow cytometry. In all statistical plots, data are expressed as the mean ± SD, One‐way ANOVA (B,C) Two‐way ANOVA (F,N,R,W) and Student's *t*‐test (H,X) were used to determine statistical significance. (ns = not significant, *p < 0.05, **p < 0.01, ***p < 0.001, ****p < 0.0001).

Based on these findings, we further investigated the link between RNF32 overexpression and SPP1⁺ macrophages to validate their role in CRC liver colonization. For in vivo validation, macrophages were depleted in mice using clodronate liposomes (CLD). In the liver metastasis model, the RNF32+CLD group showed a significant reduction in metastatic tumor nodules and liver weight compared to the RNF32 overexpression group (**Figure** **7**A,B). Immunofluorescence analysis indicated that RNF32 overexpression enhanced F4/80 expression, which was attenuated upon CLD‐mediated macrophage depletion (Figure 7C,D). We further examined the effect of conditioned medium from RNF32‐overexpressing CRC cells on macrophages in vitro using both human (THP‐1) and mouse (BMDM) macrophage models. Immunofluorescence and flow cytometry analyses revealed that exposure to RNF32‐overexpressing cancer cell supernatant promoted an M2‐like phenotype, characterized by increased CD206 and decreased CD86 expression (Figure S5A–D, Supporting Information). Additionally, RNF32‐overexpressing conditioned medium promoted SPP1 expression in THP‐1 macrophages, which was confirmed by both immunofluorescence and flow cytometry (Figure 7E–H). This finding was reinforced by immunofluorescence staining showing the same upregulation of SPP1 in human monocyte‐derived macrophages (hMDMs) differentiated from peripheral blood monocytes (PBMCs) (Figure S5E, Supporting Information). To explore how RNF32 upregulates SPP1 in macrophages, we considered its known role in activating the Wnt pathway, which has been linked to CCL2 upregulation.^[^ ^22^ ^]^ Western blot analysis showed that RNF32 overexpression enhanced CCL2 expression, an effect reversible by the Wnt inhibitor XAV‐939, suggesting Wnt pathway dependency (Figure 7I). Furthermore, RNF32‐overexpressing metastases were enriched in a distinct population of macrophages characterized by high expression of CCR2 (the CCL2 receptor) and FABP1 (Figure 7J–K). Given our prior finding that FABP1 modulates PPARG during tumor progression.^[^ ^23^, ^24^ ^]^ We hypothesized that CCL2 promotes SPP1 expression via the FABP1/PPARG axis. Western blot and immunofluorescence confirmed that CCL2 supplementation or FABP1 overexpression increased PPARG and SPP1 levels (Figure 7L–N), with co‐localization of FABP1 and PPARG observed (Figure S5F, Supporting Information). To further delineate the role of CCL2 in macrophage polarization, we treated hMDMs with increasing concentrations of recombinant CCL2. This treatment induced a clear dose‐dependent upregulation of the M2 markers CD206 and SPP1 (Figure S5G, Supporting Information), establishing CCL2 as a sufficient driver of this phenotypic shift. The functional importance of the CCL2‐CCR2 axis was confirmed using the CCR2 inhibitor RS504393, which partially reversed the FABP1‐induced upregulation of PPARG and SPP1 (Figure 7O). In addition, RS504393 partially attenuated the upregulation of CD206 and SPP1 in hMDMs induced by conditioned medium from RNF32‐overexpressing cancer cells, as visualized by immunofluorescence (Figure S5E, Supporting Information). To elucidate how the FABP1/PPARG axis regulates SPP1, we analyzed several transcription factor databases (JASPAR, CHEA, GTRD, ChIPBase) and identified PPARG as a potential direct transcriptional regulator of SPP1 (Figure 7P). JASPAR‐based prediction revealed two putative PPARG binding sites in the SPP1 promoter region (Figure 7Q). Dual‐luciferase reporter assays with site‐directed mutagenesis demonstrated that mutation at site 1 abolished PPARG‐driven SPP1 transactivation (Figure 7R), indicating that PPARG directly stimulates SPP1 transcription by binding to its promoter. We next assessed the spatial association between SPP1⁺ macrophages and cancer stem cells (CSCs) in clinical and murine CRC liver metastases. Immunofluorescence staining revealed close proximity between SPP1⁺ macrophages and CD44⁺ CSCs within tumors (Figure 7S,T). To determine whether FABP1⁺SPP1⁺ macrophages enhance cancer stemness, we co‐cultured FABP1‐overexpressing THP‐1 macrophages with HCT116 cells. Western blot analysis showed upregulation of Wnt pathway activity and stemness markers (CD44, LGR5) in tumor cells, which was partially reversed by the FABP inhibitor orlistat (Figure 7U). Immunofluorescence and flow cytometry further confirmed that FABP1‐overexpressing SPP1⁺ macrophages promote a stem‐like phenotype in CRC cells (Figure 7V–X), consistent with prior reports of SPP1⁺ macrophages supporting cancer stemness.^[^ ^25^ ^]^ In summary, our study demonstrates that RNF32 promotes CRC liver metastasis by shaping an immunosuppressive microenvironment enriched with SPP1⁺ macrophages, which in turn enhances tumor stemness.

### 2.8 A Therapeutic Redemption: Indole‐3‐Acetic Acid Targets RNF32 to Avert Metastatic Progression

**Figure 8:**
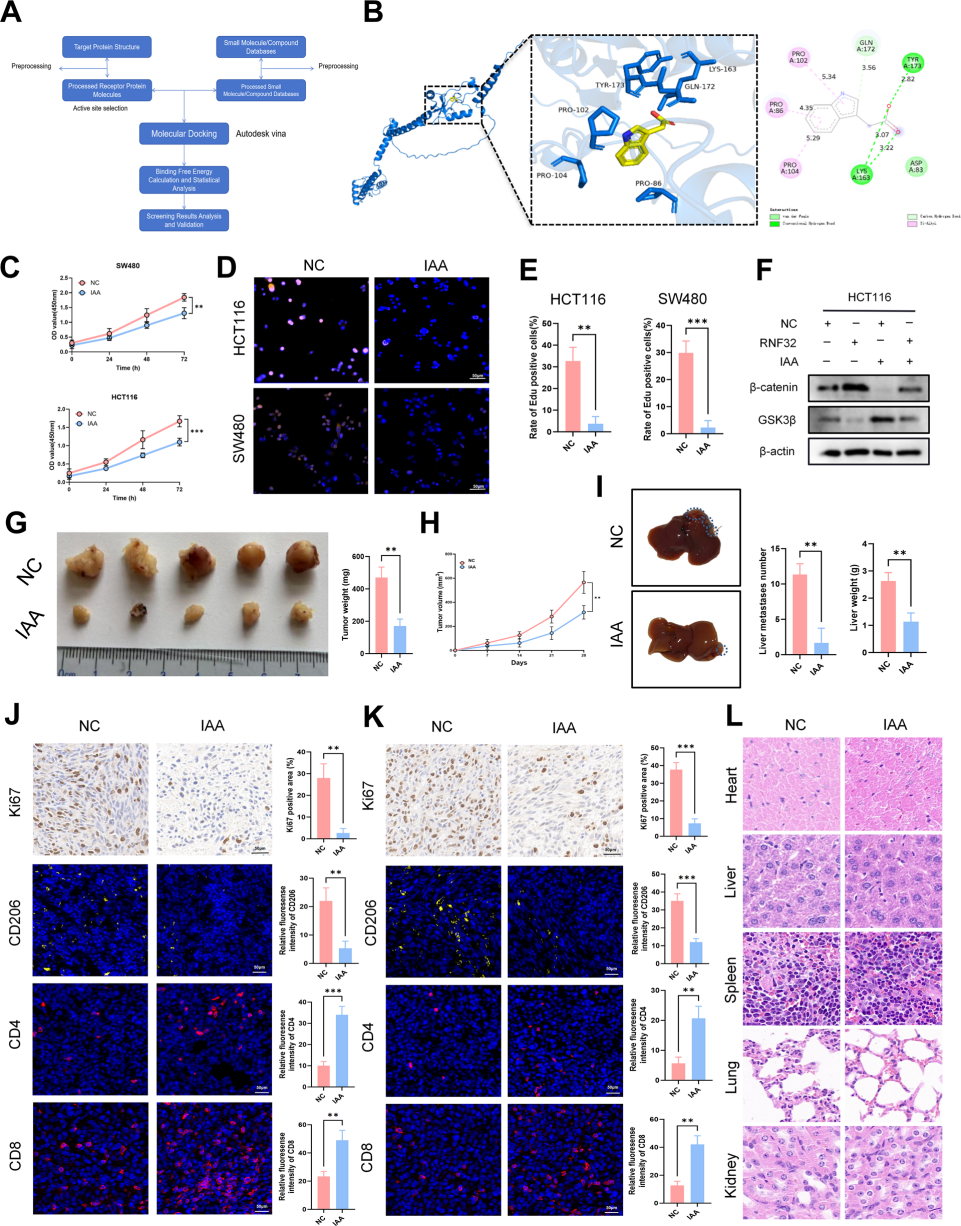
IAA, a novel RNF32 inhibitor, suppresses CRC growth and liver metastasis. A) Flowchart of virtual screening. B) 2D and 3D patterns of IAA and Human RNF32 binding. C–E) CCK‐8 and EdU assays were carried out to assess the effect of IAA (scale bars, 50 µm) on CRC cell proliferation. n = 3 independent biological replicates. F) Western blot analysis the protein levels of β‐catenin and GSK3β in HCT116 cells transfected with or without RNF32, as well as cells treated with or without IAA. β‐actin was used as the loading control. n = 3 independent biological replicates. G) Graphs of xenograft tumor weights. n = 5 mice per group. H) Growth curves of xenograft tumor volumes. n = 5 mice per group. I) The number and liver weight of the liver metastases of mice (n = 5 mice per group). J) Representative Ki‐67 immunohistochemical and CD4, CD8, CD206 immunofluorescence staining of xenograft tumors (scale bars, 50 µm). n = 3 mice per group. K) Representative Ki‐67 immunohistochemical and CD4, CD8, CD206 immunofluorescence staining of liver metastases (scale bars, 50 µm). n = 3 mice per group. L) HE staining picture of respective mice organs or tissues treated with IAA or not (scale bars, 50 µm). In all statistical plots, data are expressed as the mean ± SD, Student's *t*‐test (E,G,I,J,K) were used to determine statistical significance. (**p < 0.01, ***p < 0.001).

Recent studies indicate limited exploration of RNF32's functional roles and a scarcity of reported small‐molecule drugs targeting it. To identify potential RNF32 inhibitors, we performed virtual screening against the human RNF32 protein using the Virtual Screening Workflow module combined with Glide docking (**Figure** **8**A). The top five candidate compounds included Omitapide, Dutasteride, Ergotamine, Olaparib, and IAA (Figure S6A, Supporting Information). Among these, IAA demonstrated the highest binding affinity to RNF32, forming key interactions with residues Pro86, Pro102, Pro107, Tyr174, Lys163, and Gln172 on Chain A (Figure 8B). Using MST, we determined the binding affinity between IAA and the RNF32 protein. The results indicate that IAA binds to RNF32 with a dissociation constant (Kd) of 41.281 µM (Figure S6A, Supporting Information). Treatment of CRC cells with IAA significantly suppressed cell proliferation (Figure 8C–E). However, IAA does not affect macrophage polarization (Figure S6B, Supporting Information). Western blot analysis suggested that IAA exerts its effects by targeting RNF32 and modulating the Wnt pathway (Figure 8F). In vivo subcutaneous tumor experiments further confirmed that IAA inhibits CRC tumor growth (Figure 8G,H). Using a spleen–liver metastasis model, we observed that IAA treatment markedly reduced both the number of liver metastases and liver weight (Figure 8I). Immunohistochemical and immunofluorescence staining of subcutaneous tumors and liver metastatic foci revealed decreased expression of Ki67 and CD206, along with increased levels of CD4 and CD8 in IAA‐treated groups (Figure 8J,K). Notably, hematoxylin and eosin (HE) staining showed that IAA did not induce detectable toxicity in major organs, including the heart, liver, spleen, lungs, and kidneys of treated mice (Figure 8L). In summary, virtual screening identified IAA as a high‐affinity binder of RNF32. IAA significantly inhibited CRC proliferation in vitro, and suppressed both subcutaneous tumor growth and liver metastasis in vivo, supporting its potential as a therapeutic agent targeting RNF32 in CRC.

## 3 Discussion

The high propensity for metastasis is a major determinant of the elevated mortality rate in CRC.^[^ ^26^ ^]^ CRC frequently metastasizes to distant organs, with the liver being the most common site; ≈20% of patients already present with liver metastases at initial diagnosis.^[^ ^27^ ^]^ Liver metastasis in CRC is a multi‐step, highly coordinated biological process. Key steps include malignant transformation at the primary site, EMT‐driven invasion, intravasation of tumor cells into the circulation to form circulating tumor cells (CTCs), CTC survival in the bloodstream through immune evasion and metabolic adaptation, extravasation and colonization within the liver microenvironment, and eventual metastatic outgrowth via microenvironmental remodeling.^[^ ^28^, ^29^ ^]^ A deeper understanding of the characteristics of liver metastases is therefore critical for improving overall management of CRC patients with hepatic spread.

In this study, through integrated bioinformatics, functional validation, and mechanistic investigation, we identify RNF32 as a pivotal driver of CRC liver metastasis. Our multi‐omics analysis of TCGA data, employing rigorous Cox regression and WGCNA, pinpointed RNF32 as the sole overlapping gene significantly associated with both poor prognosis and distant metastasis. Single‐cell data further indicated significant enrichment of RNF32 within tumor cells. This systematic filtering underscores RNF32's specific role in hepatic dissemination, beyond its potential involvement in general CRC progression. RING finger proteins, characterized by a RING finger motif, represent a subclass of zinc finger proteins. They function as E3 ubiquitin ligases, facilitating the binding of E2 ubiquitin‐conjugating enzymes to specific substrates, thereby catalyzing target protein ubiquitination and subsequent proteasomal degradation. Accumulating evidence confirms that many RNF proteins possess E3 ligase activity, with their involvement in ubiquitination pathways representing a core biological function.^[^ ^10^, ^11^ ^]^ However, research focused specifically on RNF32 remains very limited, particularly at the experimental level. Our work helps to fill this gap by revealing RNF32 as a potential novel target in CRC liver metastasis. Mechanistically, we uncover a novel ubiquitin‐dependent pathway governing EMT. We demonstrate that RNF32, acting as an E3 ubiquitin ligase, directly binds to GSK3β, catalyzing its ubiquitination and proteasomal degradation. This leads to stabilization and nuclear translocation of β‐catenin, resulting in transactivation of Wnt target genes. To our knowledge, this is the first study to show RNF32‐mediated ubiquitination of GSK3β—a central negative regulator of the Wnt pathway—thereby establishing a previously unrecognized layer of post‐translational control in metastatic EMT. While the Wnt/GSK3β pathway is well‐established in CRC EMT, as shown in studies reporting roles for ADAMDEC1,^[^ ^30^ ^]^ KLHL22,^[^ ^31^ ^]^ and miR‐29c,^[^ ^32^ ^]^ our findings provide a novel molecular mechanism underlying EMT in CRC liver metastasis centered on RNF32.

Beyond this cell‐autonomous role in EMT, we elucidate how RNF32 orchestrates hepatic colonization via systemic immune reprogramming. Mass cytometry analysis of liver metastases revealed that RNF32 overexpression remodels the metastatic niche, depleting immunostimulatory CD8⁺ and CD4⁺ T cells and NK cells, while expanding SPP1⁺ macrophages, fibroblasts, and immunosuppressive monocytes. Notably, our research delineates the mechanism by which the CCL2/FABP1/PPARG axis promotes SPP1 expression in macrophages. A growing body of evidence highlights the critical role of SPP1⁺ macrophages in tumor progression. These macrophages, which express osteopontin (encoded by the SPP1 gene), are not passive markers but active accomplices within the tumor microenvironment, driving malignancy through multiple mechanisms. Studies have shown that SPP1 can directly inhibit the anti‐tumor activity of T cells and that SPP1⁺ macrophages typically exhibit an M2‐like, pro‐tumor phenotype, secreting immunosuppressive cytokines (e.g., IL‐10, TGF‐β) and recruiting regulatory T cells (Tregs) to collectively suppress CD8⁺ and CD4⁺ T cell function.^[^ ^33^, ^34^, ^35^ ^]^ SPP1 can also act as a chemoattractant, recruiting more myeloid‐derived suppressor cells (MDSCs) into the tumor microenvironment.^[^ ^36^, ^37^ ^]^ In pancreatic cancer, research has shown that SPP1 can promote cancer stemness via CD44.^[^ ^25^ ^]^Consistent with this, our study found that SPP1⁺ macrophages are located in close proximity to CD44⁺ CRC cells in vivo, and in vitro experiments further confirmed their role in promoting tumor stemness. These findings suggest that SPP1⁺ macrophages collaborate intimately with tumor cells to drive the development and progression of CRC liver metastasis.

**Figure 9:**
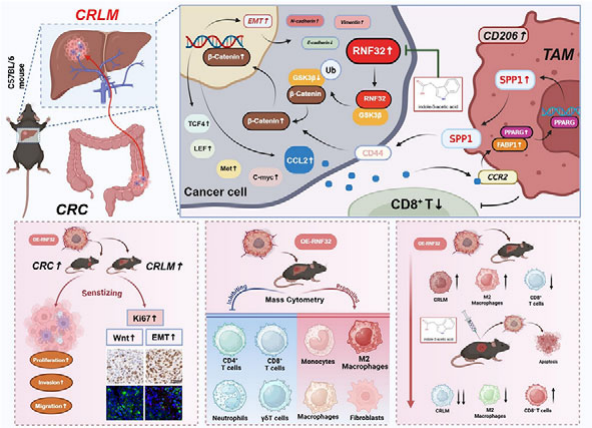
Flowchart of the mechanism. In CRC cells, cytoplasmic RNF32 directly binds to and catalyzes the ubiquitination of GSK3β, targeting it for proteasomal degradation. This process stabilizes β‐catenin, promotes its nuclear translocation, and consequently activates the Wnt signaling pathway, thereby triggering epithelial‐mesenchymal transition (EMT) to facilitate hepatic metastasis seeding. Furthermore, RNF32 overexpression remodels the metastatic niche by reducing the infiltration of CD8^+^/CD4^+^ T cells and NK cells, while simultaneously recruiting SPP1^+^ macrophages, fibroblasts, and immunosuppressive monocytes to collectively promote liver metastasis colonization. These recruited SPP1^+^ macrophages enhance tumor cell stemness via CD44. Importantly, indole‐3‐acetic acid (IAA) treatment effectively targets RNF32, reversing both tumor malignancy and the immunosuppressive microenvironment.

From a therapeutic perspective, we bridge mechanistic insight to translational application. Virtual screening identified (IAA) as a novel RNF32 inhibitor, which effectively suppressed CRC proliferation in vitro and attenuated both subcutaneous tumor growth and liver metastasis in vivo. Remarkably, IAA treatment reversed the RNF32‐associated immunosuppressive landscape, increasing CD4⁺/CD8⁺ T cell infiltration while reducing macrophage markers and proliferative activity in metastases. IAA, widely recognized as a plant growth hormone, is also present in mammals where it can be produced by gut microbiota. Contemporary research has revealed IAA as a multifaceted compound with significant influence over various physiological and pathological processes.^[^ ^38^ ^]^ For instance, Joseph Tintelnot et al. reported that microbiota‐derived IAA can influence chemotherapy efficacy in pancreatic cancer;^[^ ^39^ ^]^ Shuning Zhang et al. revealed that intestinal crypt microbiota modulates intestinal stem cell turnover and tumorigenesis via IAA;^[^ ^38^ ^]^ and Juanjuan Wang et al. demonstrated that gut microbiota‐derived IAA ameliorates precancerous colon inflammation via IL‐35, thereby inhibiting tumorigenesis.^[^ ^40^ ^]^ Our study provides both a novel therapeutic strategy and a theoretical foundation for using IAA to treat CRC liver metastasis, highlighting its dual potential in suppressing tumor cells and normalizing the tumor microenvironment **Figure** **9**.

## 4 Conclusion

This study delineates RNF32 as a master regulator of CRC liver metastasis through two synergistic mechanisms: first, as an E3 ubiquitin ligase that degrades GSK3β via ubiquitination, stabilizing β‐catenin to drive EMT‐ representing the inaugural report of post‐translational Wnt control by RNF32; second, as an orchestrator of metastatic colonization through SPP1^+^ macrophage polarization, with macrophage depletion abolishing metastasis. Therapeutically, virtual screening identified IAA, as a novel RNF32 inhibitor that concurrently suppresses tumor growth and reverses immune evasion, providing a dual‐mechanism therapeutic strategy. These findings position RNF32 as both a prognostic biomarker and actionable target for liver metastatic CRC.

## 5 Experimental Section

### Bioinformatics

Determination of differentially expressed genes (DEGs): Identification of DEGs was performed using CRC patient data from The Cancer Genome Atlas (TCGA: https://portal.gdc.cancer.gov/). This included gene expression profiles and comprehensive clinicopathological characteristics: survival time (follow‐up ≥30 days), survival status, age, gender, TNM stage, T stage, M stage, and lymph node status. Among 524 TCGA specimens retrieved (483 colon cancers and 41 normal tissues), only two colon adenocarcinoma (COAD) samples lacked clinical data (Table S1, Supporting Information). The limma R package identified DEGs between primary CRC tumors and normal tissues, applying threshold parameters of *p* < 0.05 and |log_2_ fold change (FC)| > 1. Volcano plots and heatmaps delineating DEGs were generated using R software version 4.4.1.

Analysis of DEGs enriched by Gene Ontology (GO), Kyoto Encyclopedia of Genes and Genomes (KEGG), and Gene Set Enrichment Analysis (GSEA): The DAVID database 6.8 (https://david.ncifcrf.gov/) was employed for GO enrichment analysis and KEGG enrichment analysis. The gseaplot2 R package was used for GSEA enrichment analysis.

Identification of genes independently associated with CRC prognosis: Comprehensive clinicopathological data encompassing survival time, survival status, age, gender, TNM stage, T stage, M stage, and lymph node status were extracted for 309 CRC patients (retained sufficient clinical information) from the TCGA database (Table S2, Supporting Information). Prognostic analysis was conducted on this cohort using R software (version 4.4.1), employing univariate Cox regression, Least Absolute Shrinkage and Selection Operator (LASSO) regression, and multivariate Cox regression to identify genes demonstrating independent prognostic relevance in CRC.

WGCNA: Complete clinicopathological characteristics for 309 CRC patients, including survival time, survival status, age, gender, TNM stage, T stage, M stage, and lymph node stage, were obtained from the TCGA database. Subsequently, the 5093 previously identified DEGs were subjected to WGCNA using the clinical data from these 309 samples to identify genes associated with M stage. A co‐expression network was constructed using the TCGA dataset expression data and the “WGCNA” R package. A cut height of 40 000 was chosen to remove outliers. Similar genes were then clustered into modules using the optimal soft‐thresholding power. Correlations between the modules and clinical traits were calculated. Genes within modules significantly correlated (*p* < 0.05) with M stage were extracted, yielding a total of 604 genes for subsequent analysis.

Determination of key genes associated with colon cancer liver metastasis and poor prognosis: Venn diagrams were plotted using R (4.4.1) to identify the intersection between the 17 genes independently associated with colon cancer prognosis and the 604 genes obtained from WGCNA.

Kaplan‐Meier (KM) survival analysis: The quartile of the target gene expression in CRC was used as the boundary to separate high‐expression and low‐expression groups. Survival analysis via KM curves was performed using the Gene Expression Profiling Interactive Analysis (GEPIA2) server tool (http://gepia2.cancer‐pku.cn/). A *p*‐value < 0.05 was set as the significance threshold.

Validation of the relationship between RNF32 and M stage: The TCGA dataset was used as a validation cohort to explore associations between the target gene and M stage.

Validation of gene expression levels: Datasets GSE81558 and GSE49355 were downloaded from the Gene Expression Omnibus (GEO) (https://www.ncbi.nlm.nih.gov/geo/). GEO2R, an online R‐based data processing tool, was used for analysis, and GraphPad Prism 10.0 was used for data visualization (Table S3, Supporting Information).

Validation of correlation with Wnt pathway‐related markers and EMT makers: Spearman's rank analysis was employed to detect the relationship between mRNA expression levels of RNF32 and Wnt pathway‐related markers and EMT makers in the TCGA database.

Single‐cell sequencing: Following deep single‐cell RNA sequencing of primary colorectal tumors, liver metastases, and matched adjacent tissues, cells were filtered based on standard quality controls (UMI counts: 3000–40 000; mitochondrial gene content < 10%). Normalized gene expression was calculated as LOG2(UMI + 1).

### Patient and Tissue Specimen Collection

This study was conducted in accordance with the STROBE guidelines for observational research. All study procedures strictly adhered to the Declaration of Helsinki. Participants received comprehensive information regarding research protocols and objectives, with written informed consent secured from every patient before commencement. Prior approval for human specimen collection was obtained from the Medical Ethics Committee of Nanjing Medical University. The correlation analysis was performed using 66 primary colorectal cancer tissue specimens. This cohort comprised primary tumors collected from patients with or without synchronous metastasis. For immunofluorescence studies, we acquired primary CRC tissues alongside matched adjacent intestinal tissues, hepatic metastatic specimens, and corresponding normal liver tissues from CRC patients. The inclusion criteria were as follows: (1) malignant tumor confirmed by postoperative pathology as adenocarcinoma; (2) distant metastasis proven by postoperative pathology. The exclusion criteria were as follows: (1) not diagnosed by histology; (2) a second primary cancer; (3) distant metastases at sites other than the liver; (4) cases who underwent metastasectomy without primary tumor surgery.

### RNA Extraction and qRT‐PCR

Transfection efficiency evaluation for lentiviral constructs, plasmids, and RNF32‐targeting small interfering RNAs in human CRC cell lines (HCT116, SW480) and murine CRC cell line MC38 was conducted via quantitative reverse transcription polymerase chain reaction (qRT‐PCR). Following manufacturer specifications, total RNA isolation employed TRIzol reagent (Invitrogen, USA). Subsequent complementary DNA (cDNA) synthesis utilized a reverse transcription kit (Takara, Japan). Messenger RNA (mRNA) expression quantification was standardized against the endogenous reference gene β‐actin (Table S4, Supporting Information).

### Cell and Cell Culture

Human CRC cell lines (HCT116 (RRID: CVCL_0291), LOVO (RRID: CVCL_0399), SW480 (RRID: CVCL_0546), SW620 (RRID: CVCL_0547)), mouse CRC cell line MC38 (RRID: CVCL_B288) and Human macrophages (THP‐1 (RRID: CVCL_0006)) were obtained from the Cell Bank of Type Culture Collection (Chinese Academy of Sciences, China) In June 2023. We have tested all cell lines for contamination, and confirmed that there is no contamination of mycoplasma, bacteria, fungi, etc. CRC cells were maintained in DMEM medium (BI, USA) and macrophages were maintained in RPMI 1640 medium (BI, USA) supplemented with 10% fetal bovine serum (FBS; Gibco, USA) at 37 °C in a constant temperature incubator.

### Cell Transfection

The short hair RNAs (shRNAs) of RNF32 were synthesized by Transheep, China. The overexpression plasmids, including Flag‐RNF32, HA‐GSK3β, Myc‐ubiquitin and FABP1, were obtained from Transheep, China. A series of truncated or mutated plasmids were obtained from Transheep, China. The above shRNAs and plasmids were transfected by Lipofectamine 3000 (Invitrogen). The sequences of were listed in Table S4, Supporting Information.

### Cell Proliferation Assay

Experimental and control groups of SW480 and HCT116 cells were seeded in 96‐well plates at 1000 cells per well in 100 µL culture medium. Cells were exposed to 10 µL CCK‐8 solution (RiboBio, China). Microplate reader measurements (Synergy, USA) recorded absorbance at 450 nm following manufacturer guidelines at 0, 24, 48, and 72‐h intervals.

Proliferative capacity was additionally evaluated using the Cell‐Light 5‐ethynyl‐2'‐deoxyuridine (EdU) Kit (RiboBio, China). Cells were plated at 50 000 cells well^−1^ in 24‐well plates. After 24‐h incubation, cultures were treated with 50 µmol L^−1^ EdU solution for 2 h, fixed in 4% paraformaldehyde, and sequentially processed with Apollo Dye Solution and DAPI per manufacturer protocols. Fluorescent imaging and quantification utilized an Olympus FSX100 microscope (Olympus, Japan).

For colony formation assessment, 1 × 10^3^ cells per well from respective intervention groups were plated in 6‐well plates. Colonies were fixed with 4% paraformaldehyde (Servicebio, China) after 14‐day culture and stained with crystal violet (Beyotime Biotechnology, China). Colony enumeration was conducted using ImageJ software.

### Transwell Migration and Invasion Assays

To evaluate cellular migration and invasion capabilities, Transwell chambers (Corning, USA) were utilized. Following the manufacturer's protocol, the upper chambers were coated with Matrigel matrix (BD Biosciences, USA) for invasion assays; this coating step was omitted for migration assays. Subsequently, 200 µL of serum‐free DMEM medium was added to each upper chamber. HCT116 cells and SW480 cells (20 000 cells each per chamber) were seeded into the upper chambers and assigned to experimental or control groups. The lower chambers received 700 µL of DMEM supplemented with 10% FBS to serve as a chemoattractant for CRC cells. Chambers were then incubated under standard culture conditions for 24 h. Following incubation, the upper chambers were carefully removed. The serum‐free medium within was aspirated. Cells adherent to the underside of the membrane were fixed with 4% paraformaldehyde for 10 min, stained with crystal violet solution (Kaigen, China) for 15 min, and subsequently washed with PBS. Finally, the stained cells were imaged under a microscope, and cell counts were performed in five randomly selected fields per membrane.

### Wound Healing Assay

HCT116 and SW480 cells were seeded in 6‐well culture plates and allowed to reach confluence post‐transfection. Manual linear wounds were created in the confluent cell monolayer using a sterile 20 µL pipette tip. After washing dislodged cells with PBS, complete medium was added, and plates were incubated at 37 °C. Wound areas were photographed at 0, 24, and 48 h using an inverted microscope, with scratch width quantified. All experiments were performed in triplicate.

### RNA Sequencing

To examine global gene expression changes upon RNF32 suppression, both RNF32‐silenced and control SW480 cells were lysed using TRIzol reagent. RNA sequencing was subsequently performed by Berry Genomics (Beijing, China).

### Mass Cytometry

Tumor tissues from two experimental mouse groups (NC group and RNF32 group) were dissociated using the Miltenyi Mouse Tumor Dissociation Kit (Miltenyi Biotec, Germany). A certain amount of tumor cells were prepared for CyTOF staining. Data preprocessing was conducted using FlowJo, followed by bioinformatics analysis. CyTOF experiments were performed at PLTTECH (Plttech, China).

### Immunofluorescence and Immunohistochemistry Assays

Immunofluorescence analysis was performed on 4‐µm sections derived from the following paraffin‐embedded tissues: primary CRC tissues paired with adjacent intestinal tissues; liver metastasis tissues alongside corresponding normal liver tissues from CRC patients with liver metastases; and tumor tissues obtained from subsequent mouse experiments. Cells/sections were fixed using 4% formaldehyde (20 min, room temperature [RT]) and subsequently permeabilized with 0.05% Triton X‐100 in PBS (5 min). Blocking was performed with 2% BSA in PBS (1 h), followed by incubation with primary antibodies overnight at 4 °C. antibodies we used included E‐cadherin, N‐cadherin, Vimentin, β‐catenin, GSK3β, RNF32, CD206, SPP1, CD86, F4/80, PPARG, FABP1, CD44, LGR5, CD4, and CD8. Subsequently, Alexa Fluor‐conjugated secondary antibodies were applied (1 h, RT). Sections were then counterstained with DAPI (Servicebio, China) and imaged. For immunohistochemistry (IHC), sections (comprising mouse xenograft tumor and liver metastasis samples from various treatment groups) were incubated with primary antibodies against KI67, CD4, and CD8 overnight at 4 °C. Detection was achieved using 3,3'‐diaminobenzidine (DAB) for chromogenic development. Quantitative image analysis for both IF and IHC was carried out using ImageJ software, focusing on the measurement of positive staining areas to facilitate statistical analysis (Table S5, Supporting Information).

### Western Blotting Method

Proteins were extracted from cells using RIPA buffer (Sigma‐Aldrich, USA), separated by SDS‐PAGE, and transferred to PVDF membranes. Membranes were probed with primary antibodies against RNF32 and LGR5, E‐cadherin, N‐cadherin, Vimentin, β‐catenin, GSK3β, AXIN1, CSNK1A1, APC, TCF4, LEF, Met, c‐Myc, HA, Flag, GST, Ub, Myc, CCL2, FABP1, PPARG, SPP1 and CD44. β‐actin served as loading control for whole‐cell and cytoplasmic proteins, while LaminB was used for nuclear proteins. Horseradish peroxidase‐conjugated secondary antibodies (Sigma‐Aldrich, USA) were applied, and proteins were detected using ECL chemiluminescence (Thermo Fisher, USA) (Table S5, Supporting Information).

### Co‐Immunoprecipitation Assay

293T cell lines were transfected with designated plasmids and subsequently cultured for 24 h under standard conditions. Cellular lysis was then performed using IP lysis buffer (supplemented with protease and phosphatase inhibitor tablets, Roche) for 30 min at 4 °C. Following centrifugation at 12 000 rpm (4 °C for 15 min), the protein‐containing supernatant was collected for immunoprecipitation. The protein extract was incubated overnight at 4 °C with protein G agarose beads (Bestchrom, China) and target‐specific antibodies. The immunoprecipitated complexes were then subjected to three consecutive washes with buffers containing either 300 mM or 150 mM NaCl to remove non‐specific bindings. Finally, the protein‐bead complexes were resuspended in 2×SDS loading buffer and maintained at 4 °C prior to immunoblotting analysis.

### GST‐Fusion Pull‐Down Assay

FLAG‐tagged RNF32 expression plasmids were transfected into *Escherichia coli* BL21 (*E. coli*). FLAG‐RNF32 was purified using a Ni2+‐NTA column (Thermo Fisher Scientific, MA, USA) following expression in *E. coli*. GST fusion assays were performed as in a previous study3. Briefly, 50 µg of GST‐GSK3β or GST was mixed with glutathione Sepharose 4 B beads (Sigma, MO, USA) and incubated with 20 µg of purified Flag‐RNF32. Anti‐FLAG and GST antibodies were used to analyze protein interactions via WB.

### Bone Marrow‐Derived Macrophage Extraction and Culture

Bone marrow‐derived cells were isolated from C57BL/6 mice euthanized via cervical dislocation. Following surface sterilization with 75% ethanol, lower limbs were aseptically excised. Femurs and tibiae were dissected free of attached hair, skin, muscle, and fascial tissues. Marrow cells were flushed from bones using complete DMEM medium into centrifuge tubes. After centrifugation, the supernatant was discarded. The cell pellet was resuspended in 1 mL of red blood cell lysis solution, vigorously mixed, and centrifuged again. The supernatant was removed, and cells were reconstituted in DMEM medium. Prior to use, cells were stimulated for 5 days with 20 ng mL^−1^ M‐CSF (MedChemExpress, China).

### Isolation, Differentiation, and Characterization of Human Primary Macrophages

Human primary macrophages were obtained from the peripheral blood of healthy volunteers who provided informed consent. Briefly, peripheral blood mononuclear cells (PBMCs) were isolated from whole blood using Ficoll‐Paque PREMIUM density gradient centrifugation. CD14‐positive monocytes were then purified from PBMCs using CD14 microbeads (Miltenyi Biotec, Germany) and magnetic‐activated cell sorting (MACS) technology, with a purity exceeding 95% as confirmed by flow cytometry. To derive macrophages, the purified monocytes were seeded at a density of 1 × 10^6^ cells mL^−1^ and cultured for 5 days in complete medium supplemented with 50 ng mL^−1^ human M‐CSF (MedChemExpress, China). Following this differentiation process, the adherent cells exhibited typical macrophage morphology.

### Flow Cytometry Detection and Analysis

THP‐1 cells induced by pretreatment with 100 ng mL^−1^ PMA for 24 h or BMDMs isolated from mouse bone marrow and cultured with 20 ng mL^−1^ M‐CSF for 5 days were used as control. The cells were co‐cultured with the supernatant of human CRC cells (HCT116) or mouse CRC cells (MC38) for 48 h at 37 °C under 5% CO_2_. After the coculture, for cell surface staining, cells were incubated with fluorescently labeled antibodies for 30 min at 4 °C in flow cytometry staining buffer (Biolegend, USA). After surface staining, cells were fixed with fixation buffer (Biolegend, USA) and prepared for intracellular protein staining. Fluorescently conjugated antibodies were used as follows: Anti‐mouse CD206 antibody (Thermo Fisher Scientific, USA), anti‐mouse SPP1 antibody (Thermo Fisher Scientific, USA), anti‐mouse CD86 antibody (Thermo Fisher Scientific, USA), anti‐mouse F4/86 antibody (Thermo Fisher Scientific, USA), and anti‐human CD206 antibody (Thermo Fisher Scientific, USA), anti‐human SPP1 antibody (Thermo Fisher Scientific, USA), anti‐human PPARG antibody (Thermo Fisher Scientific, USA), anti‐human CD44 antibody (Thermo Fisher Scientific, USA), anti‐human CD206 antibody (Thermo Fisher Scientific, USA), anti‐mouse CD44 antibody (Thermo Fisher Scientific, USA), anti‐mouse SPP1 antibody (Thermo Fisher Scientific, USA), anti‐human CD86 antibody (Thermo Fisher Scientific, USA) and anti‐human FABP1 antibody (Thermo Fisher Scientific, USA),.

### Virtual Screening

The 3D structure of the protein was retrieved from the AlphaFold Protein Structure Database (https://alphafold.ebi.ac.uk/entry/Q9H0A6). Unreasonable interatomic interactions were eliminated to ensure structural stability and rationality, thereby providing a reliable protein structural model for subsequent docking simulations. From the World Drug Library (including the FDA drug library, ≈5800+ molecules), screening was performed based on drug‐likeness rules to remove molecules lacking drug‐forming potential. Concurrently, novel small‐molecule libraries with specific structural features were constructed through chemical synthesis or computer‐aided design to increase the likelihood of identifying novel active molecules. Using molecular docking software AutoDock Vina, the prepared small‐molecule library was individually docked into the protein's active pocket. During docking, the protein active site was precisely defined, and appropriate parameters (e.g., force field type, flexible residue handling) were set to simulate realistic binding modes. Each docked complex was scored using a scoring function to predict binding affinity, enabling the selection of high‐scoring potential binders. Screened molecules were ranked by docking score, with top‐ranked molecules selected for further analysis. Molecular dynamics simulations assessed the binding stability of these molecules with the protein, monitoring conformational changes and interaction energy fluctuations. Experimental validation of virtual screening results determined actual binding constants and affinities between small molecules and the protein, identifying final drug candidates with potential activity.

### Molecular Docking

Molecular docking‐a computational technique forecasting ligand‐receptor binding orientations—was utilized in this investigation through semi‐flexible docking to establish stable complexes, serving as a cornerstone methodology in structure‐based drug design for mechanistic exploration and lead compound screening. IAA (PubChem CID: 802)/GSK3β (UniProt ID: P49841) and RNF32 (UniProt ID: Q9H0A6) were docked employing AutoDock Vina 1.1.2. The compound structure originated from the PubChem database (https://pubchem.ncbi.nlm.nih.gov/), with its 3D model construction and energy minimization conducted via ChemDraw 20.0. Protein structures derived from the AlphaFold Protein Structure Database (https://alphafold.ebi.ac.uk/) underwent pre‐processing in PyMOL 2.4, involving removal of water molecules/extraneous ligands and hydrogen atom addition. AutoDock Tools 1.5.6 generated PDBQT files for simulation. Docking parameters featured maximized box dimensions enclosing the entire protein while retaining default settings, with output configured to the top 10 binding poses. The optimal binding mode was selected based on minimal binding energy and maximal cluster frequency. Results expressed as binding energy (kcal mol^−1^) reflect mean ± SD of triplicate independent runs. Visualization using PyMOL 2.4 and Discovery Studio 2019 enabled spatial analysis of ligand‐receptor interactions, complex stability, and intermolecular contacts.

### Dual Luciferase Reporter Activity Assay

Transcriptional activation of the SPP1 promoter by PPARG was evaluated using a dual‐luciferase reporter assay system (Promega, USA). Briefly, 293T cells were cultured in 24‐well plates until they reached 80% confluence. The cells were subsequently transfected with Lipofectamine 3000 (Invitrogen). Each transfection mixture included a firefly luciferase reporter construct driven by the SPP1 promoter (either wild‐type or site‐directed mutant), a thymidine kinase promoter‐Renilla luciferase plasmid as an internal control, and either a PPARG overexpression plasmid or an empty vector control. After 48 h, firefly and Renilla luciferase activities were measured sequentially following the manufacturer's instructions.

### Microscale Thermophoresis

In our experiments, we employed the Monolith NT.115 (NanoTemper Technologies GmbH, Germany) to perform microscale thermophoresis (MST) in strict accordance with the manufacturer's recommended protocols. Fluorescently labeled RNF32 was obtained from lysates of HCT116 cells expressing GFP‐RNF32. To investigate the interaction between IAA and RNF32, we transfected HCT116 cells with a GFP‐RNF32 plasmid and harvested the cell lysates 1 day post‐transfection. A concentration gradient of IAA (MedChemExpress, China), ranging from 0 to 1 mM, was added to diluted aliquots of the lysate. The lysate was diluted at a 1.5:1 ratio with MST buffer (10 mM sodium phosphate buffer, pH 7.4, 1 mM MgCl_2_, 3 mM KCl, 150 mM NaCl, and 0.05% Tween‐20) to achieve optimal fluorescence intensity. Lysates from untransfected HCT116 cells were used as a control to determine background fluorescence, which was found to be negligible in our MST system. Measurements were carried out using premium coated capillaries (NanoTemper Technologies GmbH, Germany) under excitation with a 470 nm LED. All experiments were conducted with the infrared laser set to full power and the temperature maintained at 25 °C. Fluorescence signals were normalized and fitted using the Hill equation via MO Affinity Analysis software v2.1.3 (NanoTemper Technologies GmbH, Germany).

### Animal Models

All animal procedures received authorization from Nanjing Medical University's Animal Care Committee and adhered to institutional ethical guidelines. Male C57BL/6 mice were housed under specific pathogen‐free (SPF) conditions at the university's Experimental Animal Center, with euthanasia ultimately performed via cervical dislocation. To generate the CRLM model, 2 × 10⁶ MC38 cells (encompassing NC and RNF32 variants) in single‐cell suspension were injected into the splenic lower pole of anesthetized mice. Prior anesthesia was induced by intraperitoneal administration of 0.5% sodium pentobarbital (50 mg kg^−1^), followed by weight recording. Mice were then immobilized on a surgical platform; after dermal disinfection, a 0.5‐cm longitudinal incision was created beneath the costal margin along the left axillary line. The exteriorized spleen received the cellular inoculum, with subsequent compression of the injection site using an alcohol swab. Abdominal muscle and skin layers were sutured post‐hemostasis confirmation. Postoperative recovery occurred under SPF conditions. Experimental cohorts comprised NC, RNF32, RNF32+clodronate‐filled liposomes (CLD), and IAA groups (n = 5 group^−1^). The RNF32+CLD cohort was administered 200 µL CLD intraperitoneally commencing 24 h pre‐CRLM induction, continuing triweekly throughout modeling. IAA (10 mg kg^−1^) was intraperitoneally delivered beginning on day 7 and subsequently every 48 h for 2 weeks.

### Statistical Analysis

Statistical analyses were performed using GraphPad Prism 10.0 software. Data are presented as mean ± standard deviation (SD). Two‐group comparisons employed two‐tailed Student's *t*‐tests. Variation among multiple groups was assessed by ANOVA. Survival analysis utilized Kaplan‐Meier methodology. Statistical significance was defined as *p* < 0.05.

### Ethics Approval and Consent To Participate

This study, which involves human participants, received approval from the Medical Ethics Committee of Nanjing Medical University (2023‐SRFA‐248). All in vivo animal experiments were approved by the Committee on the Ethics of Animal Experiments of Nanjing Medical University (IACUC‐2404094).

## Conflict of Interest

The authors declare no conflict of interest.

## Author Contributions

H.W., S.D., Y.X., P.C., and Y.C. contributed equally to this work. 5 first authors were listed in this manuscript. H.Y.W.,S.P.D.,Y.C.X.,P.Y.C., and Y.C. were responsible for designing the study, performing part of the experiments, as well as drafting the manuscript. Furthermore, we have 5 corresponding authors in this manuscript. J.F.S.,Y.X.Y.,X.F.Q.,W.W.T., and Y.C.Z. have contributed to the study design, data interpretation, editing, and critical revision of the manuscript. Other authors also contributed to performing part of the experiments, and data interpretation. All authors read and approved the final manuscript.

## Supporting information



Supporting Information

Supporting Information

Supporting Information

Supporting Information

Supporting Information

Supporting Information

## Data Availability

The data that support the findings of this study are available from the corresponding author upon reasonable request.
