# Supplementary material for: Colorectal Cancer Cell's Weapon: RNF32 Engages SPP1+ Macrophages to Foster Liver Metastasis, Targeted by Indole‐3‐Acetic Acid
Source: Adv Sci (Weinh). 2025 Dec 12;13(11):e19735. doi: 10.1002/advs.202519735 (PMC12931155; doi:10.1002/advs.202519735)
Supplement: Supplementary file 1 — Supporting Information [file ADVS-13-e19735-s006.docx]

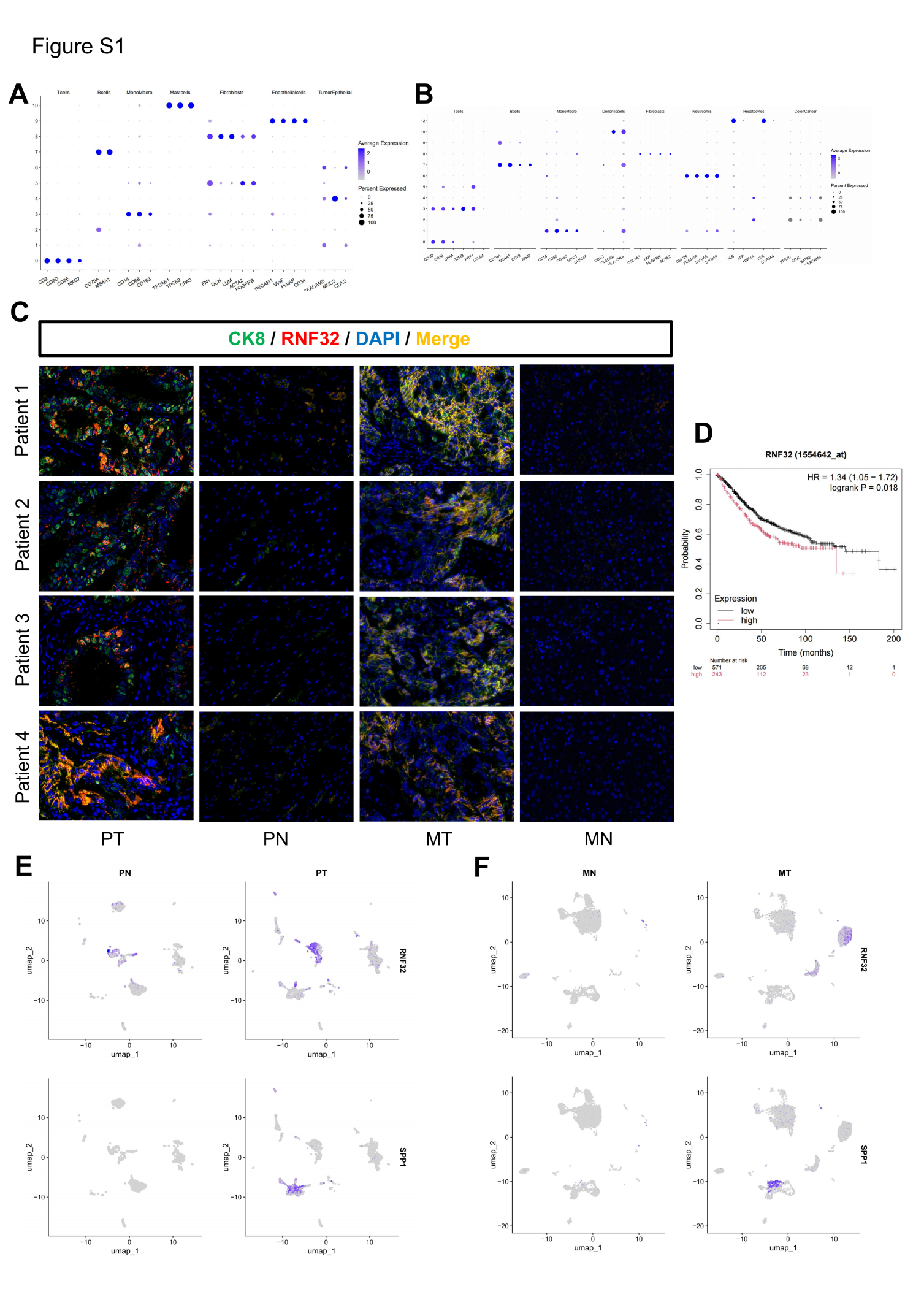


**Figure S1:**

(A-B) The dot plot shows the specific genes that define different cell clusters in Figure 1W-X

(C) Immunofluorescence confirmed high RNF32 expression in tumor tissues with co-staining for CK8 .

(D) High RNF32 levels correlated with poor prognosis.

(E-F) The UMAP plot shows the detailed distribution of RNF32 and SPP1 of each cell cluster.


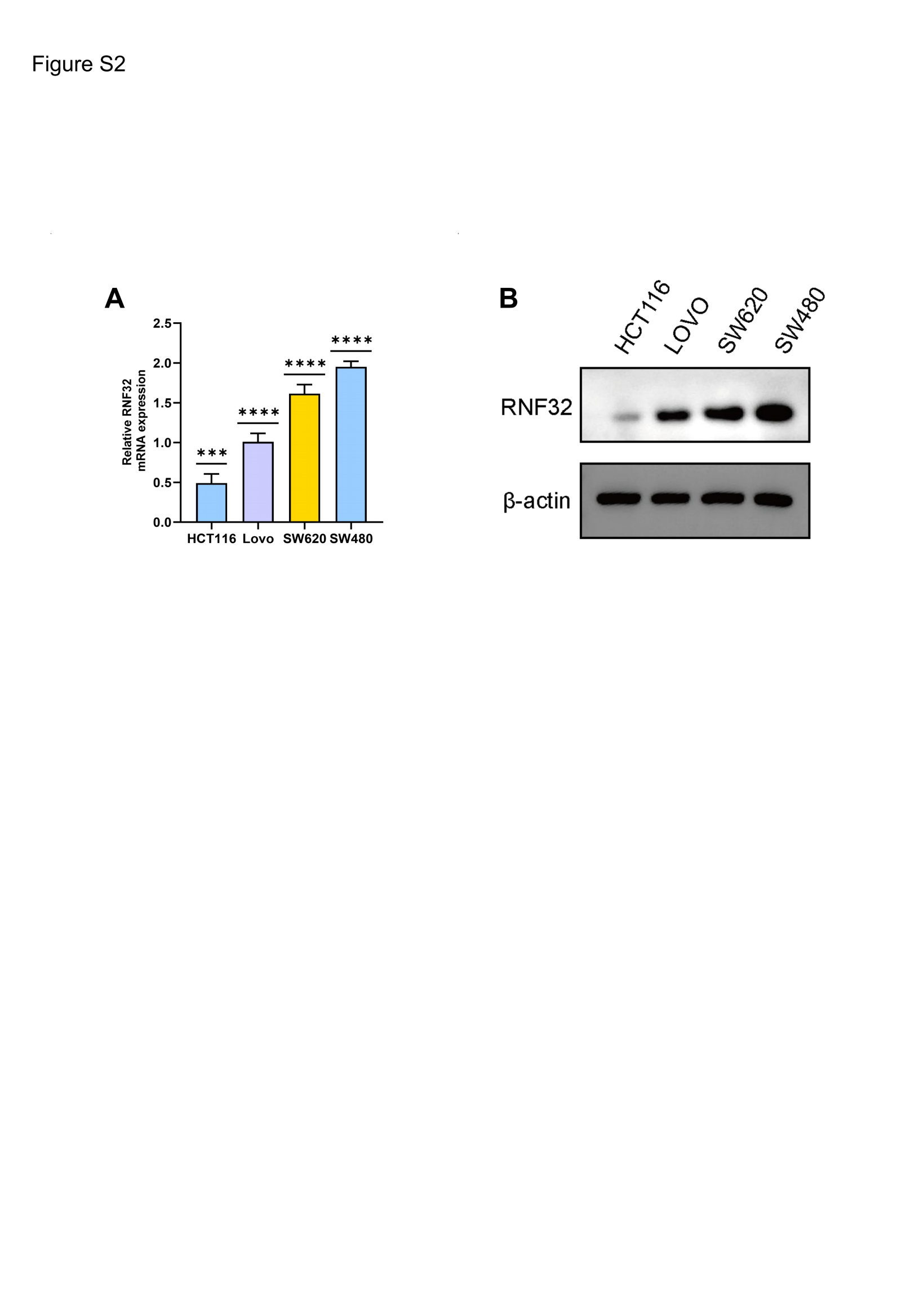


**Figure S2:**

1. Relative mRNA expression levels of RNF32 in CRC cell lines after transfection. n = 3 independent biological replicates.
2. Protein expression levels of RNF32 in CRC cell lines after transfection. β-actin is used for loading control. n = 3 independent biological replicates.

In all statistical plots, data are expressed as the mean ± SD, One-way ANOVA (Figure S2A) were used to determine statistical significance. (ns=not significant, *P < 0.05, **P < 0.01, ***P < 0.001).

**
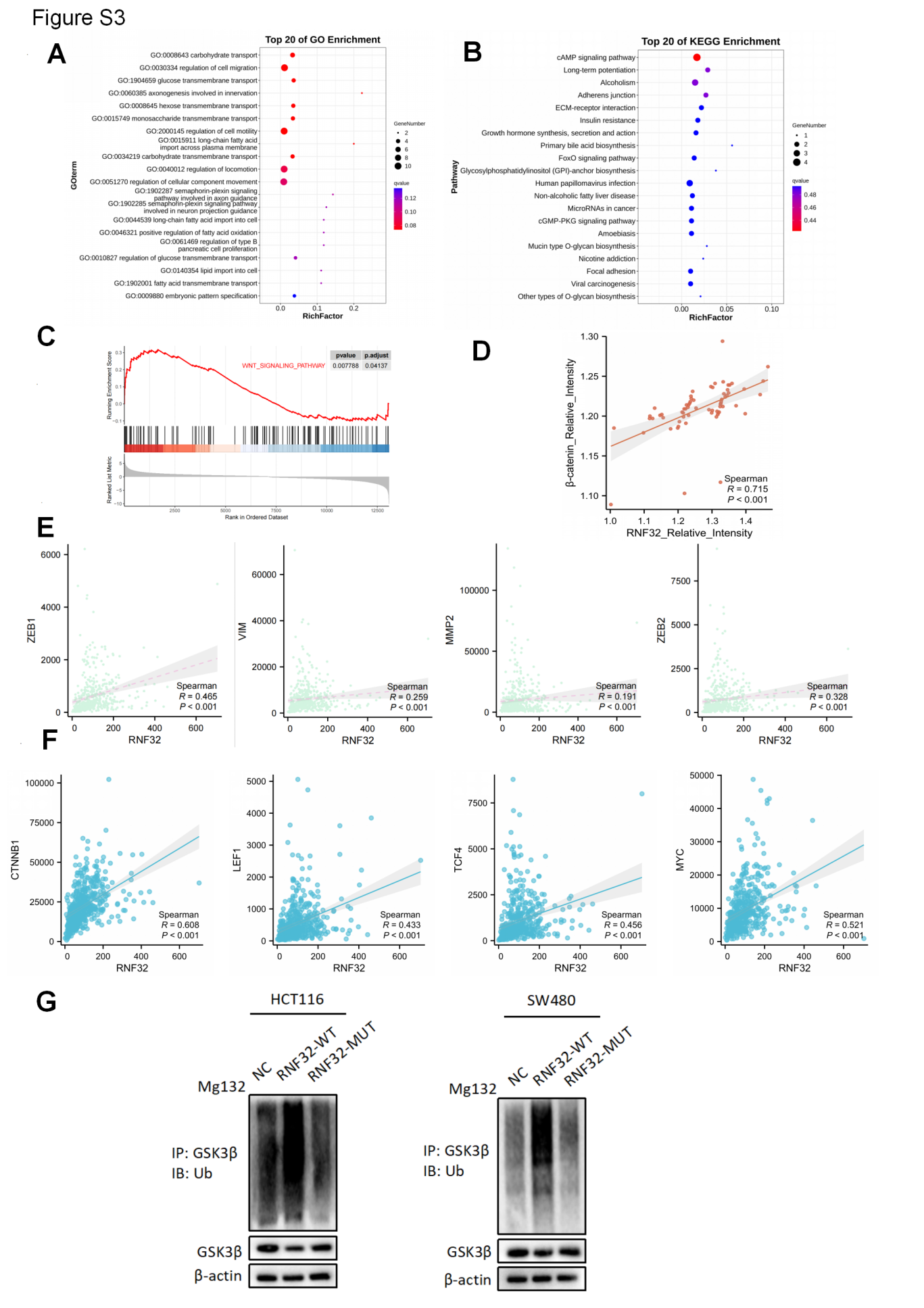
**

**Figure S3:**

(A-B) According to the results of GO analysis, differentially down-expressed genes were largely enriched in carbohydrate transport and regulation of cell migration. As revealed by KEGG analysis results, cAMP signaling pathway and long-term potentiation were among the most obviously involved processes.

(C) The enrichment plot of WNT signaling pathway by GSEA analysis in colon cancer from TCGA.

(D) Use Spearman's rank correlation analysis to examine the correlation between RNF32 and β-catenin protein expression in 66 CRC clinical samples

(E) Using Spearman’s rank analysis to examine the correlation between RNF32 and EMT-related markers in the TCGA COAD dataset.

(F) Using Spearman’s rank analysis to examine the correlation between RNF32 and the markers related with Wnt path way markers in the TCGA COAD dataset.

(G) The ubiquitination level of GSK3β in HCT116 and SW480 cells transfected with RNF32-WT and RNF32-MUT

**
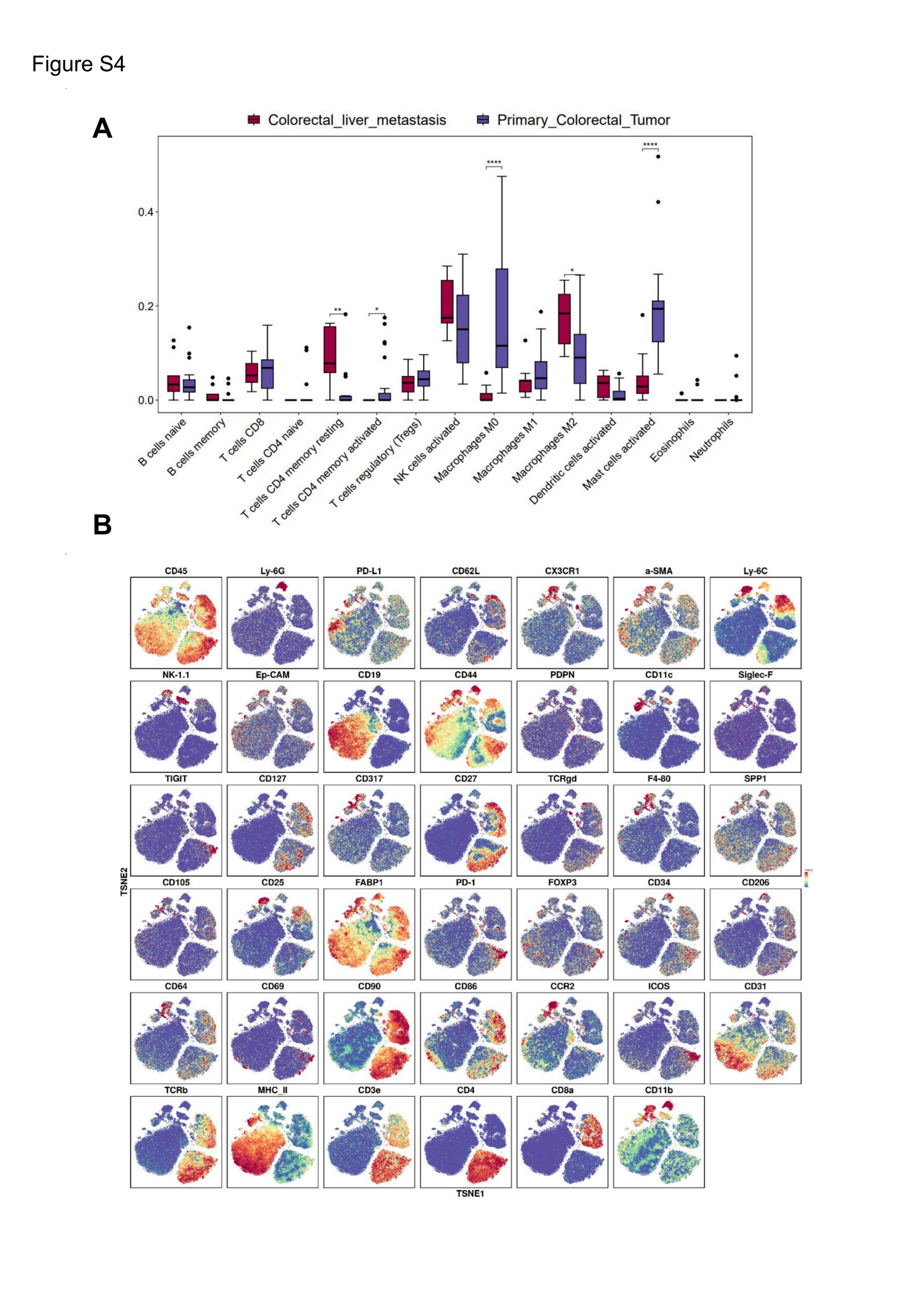
**

**Figure S4:**

1. Immune infiltration analysis of Timer dataset.
2. Liver metastases samples from mice injected with NC and RNF32 were detected subcutaneous by mass cytometry. 38 cell clusters were detected.

**
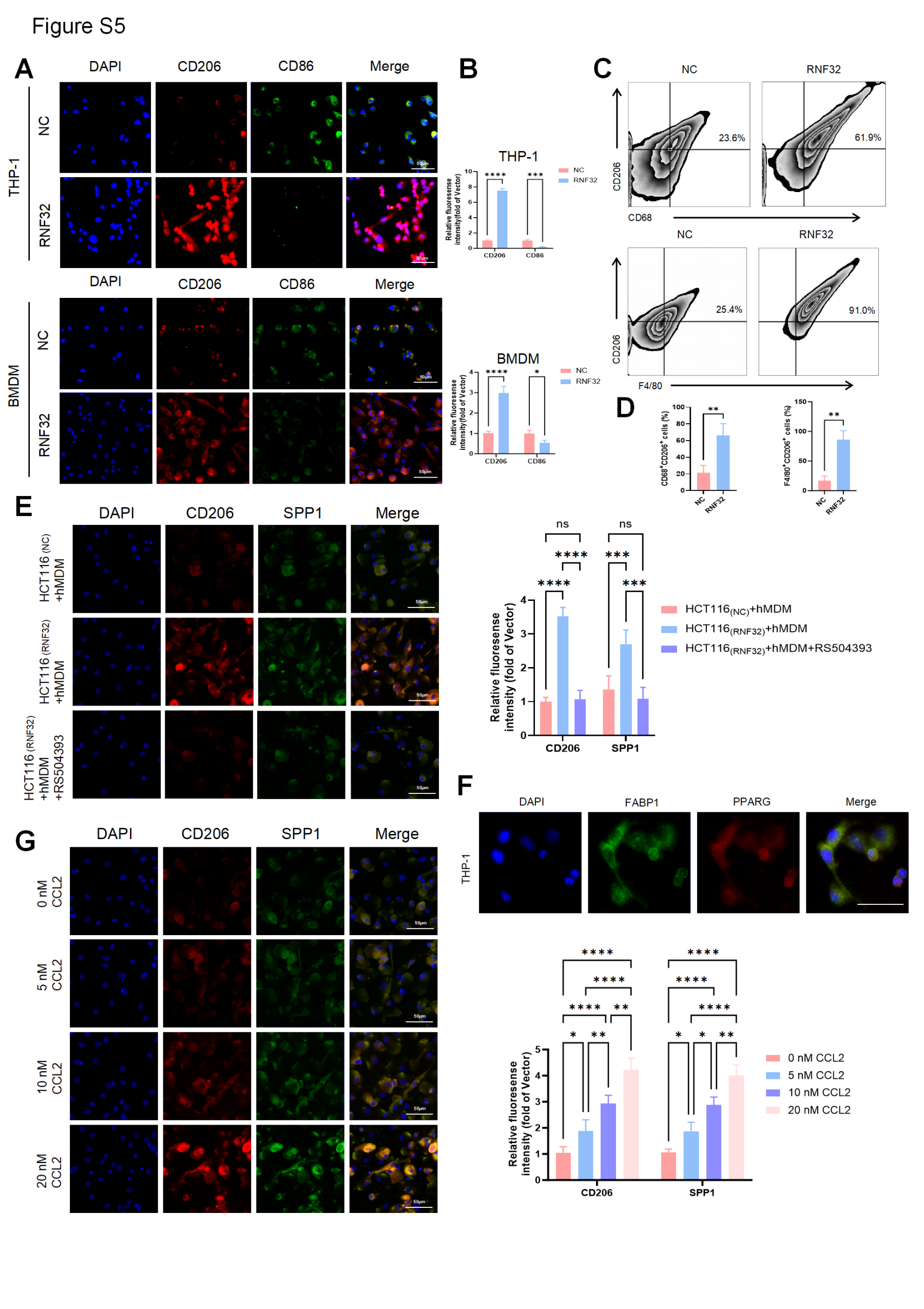
**

**Figure S5:**

(A-B) Representative CD206 and CD86 immunofluorescence staining in THP-1 and BMDM cells transfected with RNF32 (scale bars, 50μm). n=3 independent biological replicates.

(C-D) The proportions of CD68^+^CD206^+^ and F4/80^+^CD206^+^cells were analyzed separately by flow cytometry.

(E) Representative immunofluorescence staining of CD206 and SPP1 when hMDMs are cocultured with HCT116 cells overexpressing or not overexpressing RNF32, with or without the addition of RS504393 (scale bars, 50μm). n=3 independent biological replicates.

(F) Co-localization of FABP1 (green) and PPARG (red) was indicated by immunofluorescence confocal microscopy. DAPI was used for nuclear staining (blue).

(G) Representative CD206 and SPP1 immunofluorescence staining in hMDMs treated with increasing concentrations of recombinant CCL2 (scale bars, 50μm). n=3 independent biological replicates.

In all statistical plots, data are expressed as the mean ± SD, Two-way ANOVA (Figure S5B, E, G) and One-way ANOVA (Figure S5D)were used to determine statistical significance. (ns=not significant, *P < 0.05, **P < 0.01, ***P < 0.001, ****P < 0.0001).

**
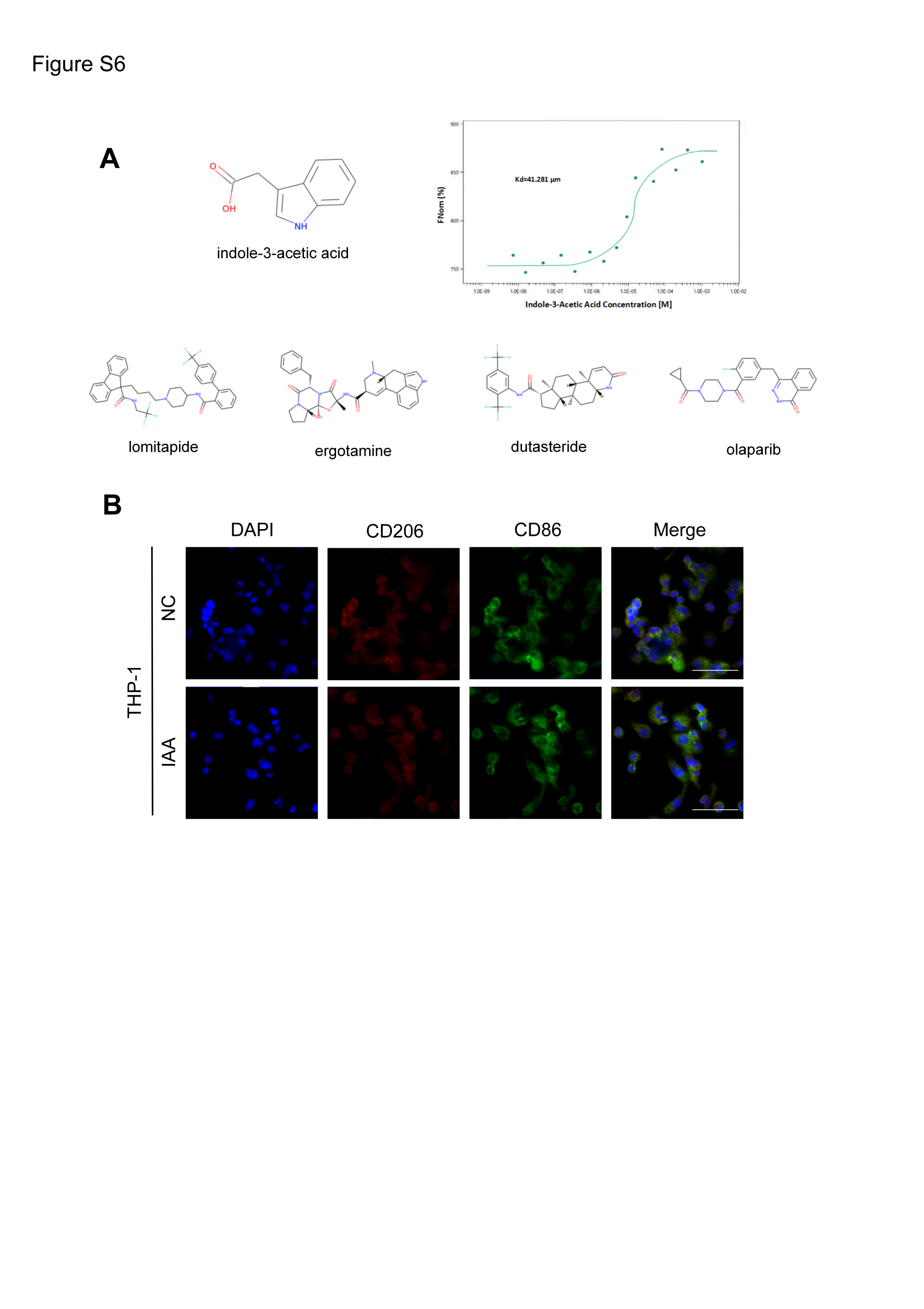
**

**Figure S6:**

1. Top five ranked compound molecular structures from virtual screening; MST analysis quantified the binding tenacity between Indole-3-Acetic Acid and RNF32.
2. Representative CD206 and CD86 immunofluorescence staining in THP-1 macrophages treated with or without IAA (scale bars, 50μm). n=3 independent biological replicates.
