# Supplementary material for: Colorectal Cancer Cell's Weapon: RNF32 Engages SPP1+ Macrophages to Foster Liver Metastasis, Targeted by Indole‐3‐Acetic Acid
Source: Adv Sci (Weinh). 2025 Dec 12;13(11):e19735. doi: 10.1002/advs.202519735 (PMC12931155; doi:10.1002/advs.202519735)
Supplement: Supplementary file 6 — Supporting Information [file ADVS-13-e19735-s004.docx]

| **Antibody** | **Manufacturer** | **Application** |
| --- | --- | --- |
| RNF32 | Thermo Fisher Scientific: H00140545-B01P | 1:1000 for WB |
| RNF32 | Thermo Fisher Scientific: PA5-139766 | 1:500 for IF |
| GSK3β | proteintech:82061-1-RR | 1:2000 for WB, 1:100 for IF |
| E-cadherin | proteintech:20874-1-AP | 1:20000 for WB, 1:800 for IF |
| N-cadherin | proteintech:22018-1-AP | 1:2000 for WB, 1:800 for IF |
| Vimentin | proteintech:10366-1-AP | 1:20000 for WB, 1:800 for IF |
| β-catenin | proteintech:51067-2-AP | 1:5000 for WB |
| AXIN1 | proteintech:16541-1-AP | 1:1000 for WB |
| CSNK1A1 | proteintech:68434-1-IG | 1:5000 for WB |
| APC | proteintech:98136-1-RR | 1:2000 for WB |
| TCF4 | proteintech:22337-1-AP | 1:5000 for WB |
| LEF1 | proteintech:14972-1-AP | 1:4000 for WB |
| c-Met | proteintech:25869-1-AP | 1:2000 for WB |
| c-Myc | proteintech:10828-1-AP | 1:2000 for WB |
| HA | proteintech:51064-2-AP | 1:5000 for WB |
| Flag | proteintech:66008-4-Ig | 1:5000 for WB |
| GST | proteintech:10000-0-AP | 1:1000 for WB |
| Ub | proteintech:10201-2-AP | 1:1000 for WB |
| Myc | proteintech:60003-2-Ig | 1:5000 for WB |
| CCL2 | proteintech:26161-1-AP | 1:2000 for WB |
| FABP1 | proteintech:13626-1-AP | 1:2000 for WB, 1:300 for IF |
| PPARG | proteintech:16643-1-AP | 1:1000 for WB |
| SPP1 | proteintech:22952-1-AP | 1:1000 for WB, 1:200 for IF |
| CD44 | proteintech:15675-1-AP | 1:5000 for WB, 1:100 for IF |
| LGR5 | Thermo Fisher Scientific: PA5-23000 | 1:1000 for WB, 1:500 for IF |
| β-actin | proteintech:66009-1-Ig | 1:20000 for WB |
| LaminB | proteintech:12987-1-AP | 1:5000 for WB |
| CD206 | proteintech:18704-1-AP | 1:100 for IF |
| CD86 | proteintech:83523-4-RR | 1:100 for IF |
| F4/80 | proteintech:28463-1-AP | 1:100 for IF |
| CD4 | Servicebio:GB15064-100 | 1: 200 for IF/IHC |
| CD8 | Servicebio:GB15068-100 | 1: 400 for IF/IHC |
| Ki67 | Servicebio:GB111499-100 | 1: 500 for IHC |
